# Characterization of Chromosomal Breakpoints in 12 Cases with 8p Rearrangements Defines a Continuum of Fragility of the Region

**DOI:** 10.3390/ijms23063347

**Published:** 2022-03-20

**Authors:** Serena Redaelli, Donatella Conconi, Elena Sala, Nicoletta Villa, Francesca Crosti, Gaia Roversi, Ilaria Catusi, Chiara Valtorta, Maria Paola Recalcati, Leda Dalprà, Marialuisa Lavitrano, Angela Bentivegna

**Affiliations:** 1 School of Medicine and Surgery, University of Milano-Bicocca, 20900 Monza, Italy; serena.redaelli@unimib.it (S.R.); gaia.roversi@unimib.it (G.R.); leda.dalpra@unimib.it (L.D.); marialuisa.lavitrano@unimib.it (M.L.); 2 Medical Genetics Laboratory, Clinical Pathology Department, S. Gerardo Hospital, 20900 Monza, Italy; elena.sala@asst-monza.it (E.S.); n.villa@asst-monza.it (N.V.); f.crosti@asst-monza.it (F.C.); 3 Medical Cytogenetics Laboratory, Istituto Auxologico Italiano IRCCS, 20095 Cusano Milanino, Italy; ilaria.catusi@gmail.com (I.C.); citogen@auxologico.it (C.V.); p.recalcati@auxologico.it (M.P.R.)

**Keywords:** chromosome instability, cytogenetics, molecular karyotype, array CGH

## Abstract

Improvements in microarray-based comparative genomic hybridization technology have allowed for high-resolution detection of genome wide copy number alterations, leading to a better definition of rearrangements and supporting the study of pathogenesis mechanisms. In this study, we focused our attention on chromosome 8p. We report 12 cases of 8p rearrangements, analyzed by molecular karyotype, evidencing a continuum of fragility that involves the entire short arm. The breakpoints seem more concentrated in three intervals: one at the telomeric end, the others at 8p23.1, close to the beta-defensin gene cluster and olfactory receptor low-copy repeats. Hypothetical mechanisms for all cases are described. Our data extend the cohort of published patients with 8p aberrations and highlight the need to pay special attention to these sequences due to the risk of formation of new chromosomal aberrations with pathological effects.

## 1. Introduction

The implementation of microarray-based comparative genomic hybridization (array CGH) into diagnostics has introduced a new fundamental step in genetic diagnosis. This technology allows for high-resolution detection of genome-wide copy number alterations, leading to a better definition of rearrangements and helping in the study of pathogenesis mechanisms. In this study, our attention was given to 8p rearrangements; one such rearrangement, known as 8p inverted duplication/deletion syndrome (Inv dup del(8p), ORPHA:96092, https://www.orpha.net/consor/cgi-bin/OC_Exp.php?lng=EN&Expert=96092, 22 October 2021), is a recurrent structural abnormality historically recognized since the 1990s. The inv dup del(8p) consists of a deletion distal to the 8p23 region, followed by an intermediate intact segment, and a proximal inverted duplication of various extensions. These rearrangements are consistently of maternal origin and are mediated mainly by two highly repetitive and polymorphic regions: the olfactory receptor gene clusters or defensin repeat (ORDRs) [1,2,3]. The polymorphic 8p23 inversion between these clusters can mediate nonallelic homologous recombination (NAHR), increasing the susceptibility of 8p to rearrangements. The corresponding clinical phenotype includes hypotonia, developmental delay, facial dysmorphism, central nervous system abnormalities, and congenital heart defects [4].

The 8p arm is also involved in one of the most recurrent non-Robertsonian reciprocal translocations, the t(4; 8) (p16; p23), associated with some cases of Wolf-Hirschhorn syndrome (WHS) (OMIM 194190) [5,6,7]. These rearrangements usually arise during maternal meiosis as a result of NAHR mediated by olfactory receptor gene clusters on both 4p and 8p. Breakpoints usually cluster around the loci of olfactory receptor gene clusters located on both chromosomes [8].

**Figure 1:**
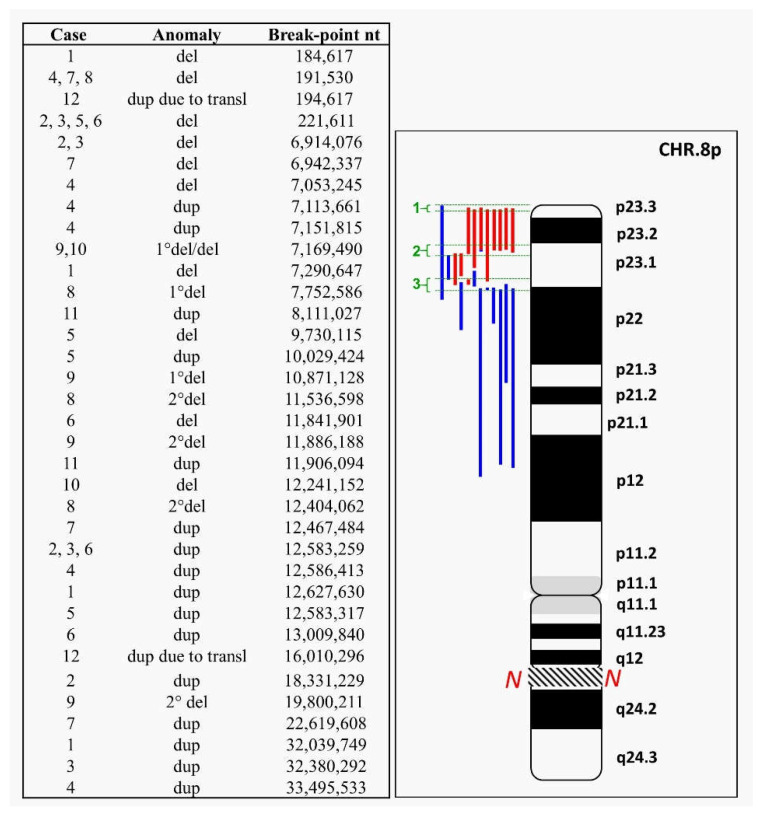
Graphical representation of chromosome 8 aberrations described in this study. Left: in the third column, from top to bottom, the identified breakpoints are reported from most distal to most proximal; in the middle, the alterations are specified as in the figure (see below); and in the first column, the cases in which they have been identified. Right: red bars: deletions (del); blue bars: duplications (dup). Green brackets show the most frequent breakpoint intervals: interval 1 is the most telomeric, in 8p23.3, from nt 184,617 to nt 221,611; intervals 2 and 3 are in 8p23.1, from nt 6,942,337 to nt 9,730,115 and from nt 10,029,424 to nt 12,627,630, respectively. The region of the 8q arm not shown in the figure is indicated by the *N*.

In this paper, we report 12 unpublished cases of 8p rearrangements, analyzed by molecular karyotype in order to improve knowledge of the mechanisms involved. We evidenced a continuum of fragility involving the entire short arm. Breakpoints seem more concentrated at three intervals: interval 1 is at the telomeric end, in 8p23.3, from nt 184,617 to nt 221,611; intervals 2 and 3 are in 8p23.1, from nt 6,942,337 to nt 9,730,115 and from nt 10,029,424 to nt 12,627,630, respectively, close to the beta-defensin gene cluster (DEFB) and olfactory receptor (OR) low-copy repeats (LCRs) [2]. A summary of the cases is shown in Figure 1.

## 2. Case Reports and Cytogenetics—Genomics Description

### 2.1. 8p Inverted Duplication with Deletion

We report seven cases with rearrangements such as inv dup del(8p).

Case 1: a 10-year-old girl with a syndromic trait.

**Figure 2:**
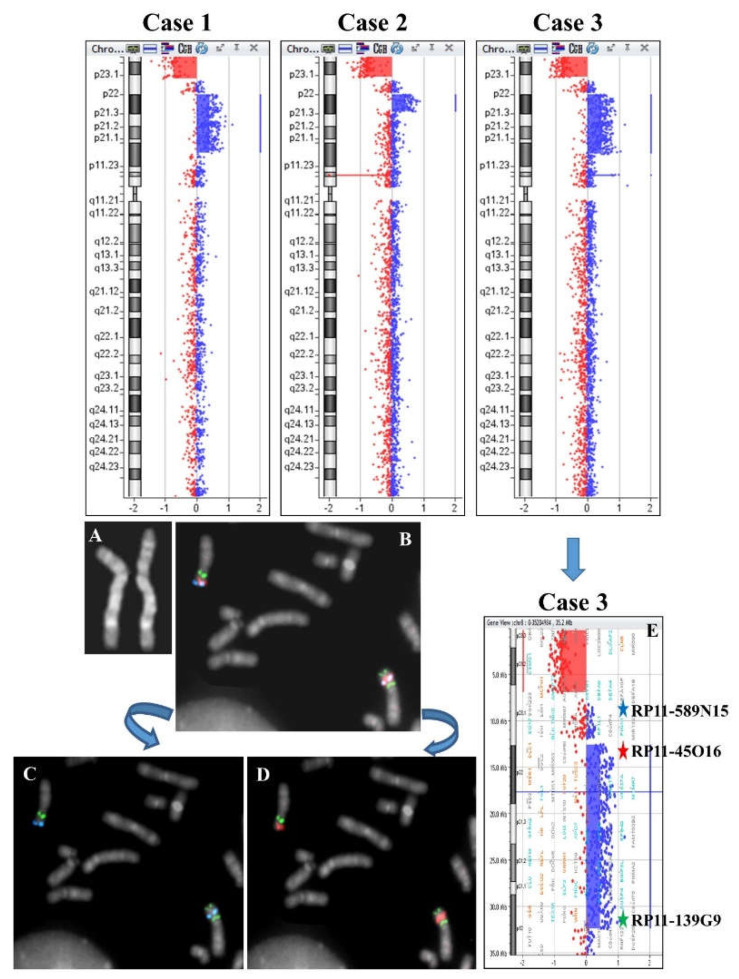
Upper panel: array CGH results of cases 1-3. Lower panel: Cytogenetics and FISH results of case 3. (**A**) QFQ-banded chromosome 8. (**B**) Partial metaphase after tricolor FISH with probes RP11-589N15 (blue signals), RP11-45016 (red signals), and RP11-139G9 (green signals). (**C**) Partial metaphase after dual-color FISH with probes RP11-589N15 (blue signals) and RP11-139G9 (green signals). (**D**) Partial metaphase after dual color FISH with probes RP11-45016 (red signals) and RP11-139G9 (green signals). (**E**) Array CGH ideogram showing the del inv dup(8p) region and the reciprocal mapping of FISH probes.

Array CGH analysis evidenced a duplication with deletion of the telomere region of 8p: arr[GRCh37] 8p23.3p23.1(184617_7290647)x1,8p23.1p12(12627630_32039749)x3. (Figure 2). The disomic tract between the deleted region and the duplicated one was 5.30 Mb.

Case 2: a 3-year-old girl with a syndromic trait.

Array CGH analysis evidenced a duplication with deletion of the telomere region of 8p: arr[GRCh37] 8p23.3p23.1(221611_6914076)x1,8p23.1p22(12583259_18331229)x3 (Figure 2). The disomic tract between the deleted region and the duplicated one was 4.09 Mb.

Case 3: a 2-year-old girl with a syndromic trait.

The karyotype evidenced an inverted duplication and deletion of 8p; the inversion was confirmed by FISH analysis (Figure 2A–E) and the deletion and duplication by array CGH: arr[GRCh37] 8p23.3p23.1(221611_6914076)x1,8p23.1p12(12583259_32380292)x3 (Figure 2). The disomic tract between the deleted and the duplicated region was 4.09 Mb.

Case 4: prenatal diagnosis for suspicion of cardiac abnormality.

**Figure 3:**
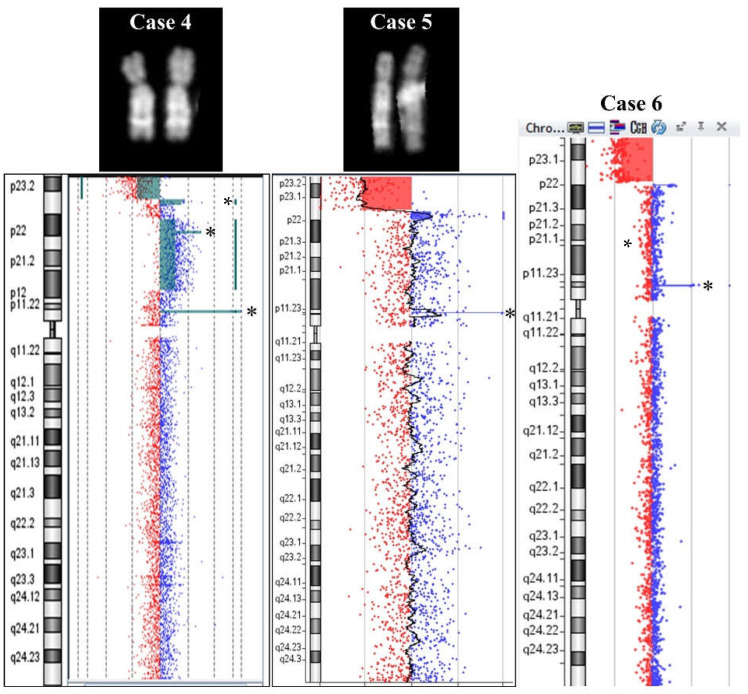
Cases 4, 5, and 6. QFQ-banded chromosomes for case 4 (**left**) and 5 (**middle**). Array CGH ideograms for case 4, 5, and 6 (from **left** to **right**). Asterisks (*) show benign CNVs.

Amniocentesis was requested at the 33rd week of gestation due to ultrasound evidence of cardiac abnormality. A conventional karyotype failed (normal result) due to the low resolution level of QFQ banding (400 bands). A subsequent molecular karyotype evidenced an abnormality of 8p: arr[GRCh37]8p23.3p23.1(191530_7053245)x1,8p23.1(7113661_7151815)x3,8p23.1p12(12586413-33495533)x3 (Figure 3). The deletion was 6.9 Mb long and the consecutive duplication was 38.2 kb. The second and longest duplication was 21 Mb. An interval of 5.5 Mb of disomic segment was present between the two duplications. Moreover, we highlighted a benign 135 kb duplication from nt 7,671,731 to nt 7,806,229 in a centromeric position with respect to the above-described smallest duplication and distant from it by more than 500 kb, probably, on the homolog 8 chromosome. One more CNV was observed in 8p11.22, which includes ADAM genes, declared benign in all consulted databases.

Case 5: prenatal diagnosis in a fetus that later died.

One couple’s first pregnancy resulted in an intrauterine fetal death at the 31st week of gestation. A conventional karyotype on cultured amniocytes evidenced female sex and a deletion on chromosome 8p: 46,XX,del(8)(p22). The parents showed normal karyotype, and FISH with subtelomeric-8-specific probes was normal for the number and position of signals. A molecular karyotype confirmed the deletion and showed a smaller duplication that was not visible with conventional cytogenetics.

The molecular karyotype was: arr[GRCh37] 8p23.3p23.1(221612_9730115)x1,8p23.1(10029424_12583317)x3 (Figure 3). The loss in 8p23.3-p23.1 was 9.6 Mb long and is defined as pathogenetic in the Troina Database (Database of Human CNVs IRCCS Oasi Maria SS Troina gvarianti.homelinux.net/gvariantib37/index.php, 22 October 2021) and the ISCA database from dbVar (www.ncbi.nih.gov/dbvar/studies/nstd102, 22 October 2021), but no overlapping alterations were found in the Database of Genomic Variants (DGV). The gain in 8p23.1 was 2.6 Mb long. The ISCA Database reports three overlapping alterations of uncertain significance, and one overlapping benign CNV was found in the Troina Database, while no overlapping CNVs were found in the DGV. No disomic interposed region was observed in this case. The molecular karyotype also evidenced a benign amplification of 127 kb at 8p11.23-p11.22.

Case 6: a 17-year-old boy with syndromic traits including intellectual disability, autism, and dysmorphisms. Only a molecular karyotype was performed: a gain was detected in the 1p terminal and a deletion together with a duplication in 8p: arr[GRCh37] 1p36.21(14933994_16100796)x3,8p23.3p23.1(221611_11841901)x1,8p23.1p22(12583259_13009840)x3 (Figure 3). Deletion and duplication of 8p seemed consecutive, without a disomic interposed region, but no probes were present in the 740 kb region between the two anomalies. The 1.2 Mb gain on chromosome 1 is of uncertain significance and includes some OMIM genes such as *KAZN* (OMIM618301), *EFHD2* (OMIM616450), *CTRC* (OMIM601405), *CELA2A* (OMIM609443), *CELA2B* (OMIM609444), *CASP9* (OMIM602234), *PLEKHM2* (OMIM609613), and *SLC25A34* (OMIM610817). Overlapping gains are described as uncertain in the ClinVar Database and in the Troina Database. No overlapping gains with a comparable size are present in the DGV. However, FAM/OR genes are absent in proximity to 1p duplication breakpoints [9].

Case 7: a 5-year-old girl with global delay and dysmorphic features.

Chromosome morphology showed an apparent deletion and duplication of 8p. FISH analysis was performed with 8p- and 8q-subtelomeric-specific probes and a cytogenetic karyotype showed the results: 46,XX,dup(8)(p21.3p23.1)del(8)(p23.1)dup(8)(q24.3).ish dup(8)(p21.3p2.1)del(8)(p23.1)dup(8) (q24.3)(D8S504-,VIJyRM2053++).

**Figure 4:**
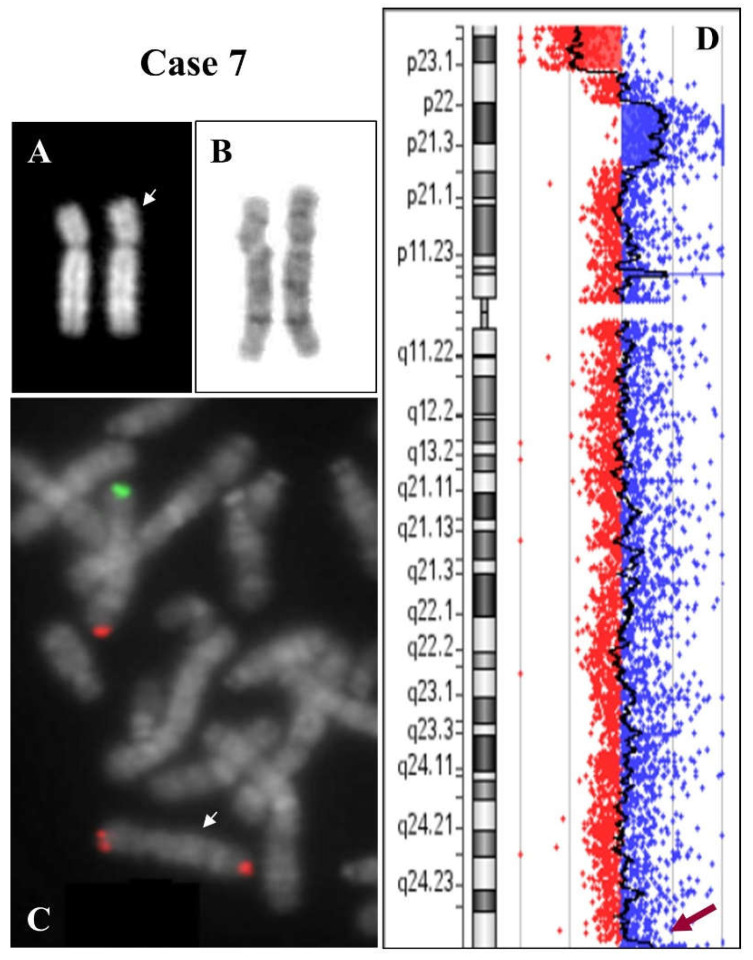
Case 7. (**A**) QFQ-banded chromosome 8 (the white arrow indicates the anomalous chromosome). (**B**) GTG-banded chromosome 8. (**C**) Partial metaphase after FISH with specific subtelomeric probes of chromosome 8 (green: telomere 8p, red: telomere 8q). The anomalous chromosome 8 shows the 8q telomere on both extremities (white arrow). (**D**) Array CGH results. The red arrow indicates a very small duplication of the 8q telomere.

A molecular karyotype confirmed and defined the conventional one: arr[GRCh37] 8p23.3p23.1(191530_6942337)x1 dn, 8p23.1p21.3(12467484_22619608)x3 dn, 8q24.3(145129398_146280020)x3 dn (Figure 4).

Moreover, a small duplication of 1.15 Mb was evidenced in the q terminal region (Figure 4C). FISH analysis showed three signals from the 8q-telomere-specific probe, confirming the data (Figure 4B). Both parents were karyotypically normal and the rearrangement on chromosome 8 in the child was de novo (dn).

### 2.2. Isolated Deletion or Duplication of 8p

We present three cases of deletion and two cases of duplication.

**Figure 5:**
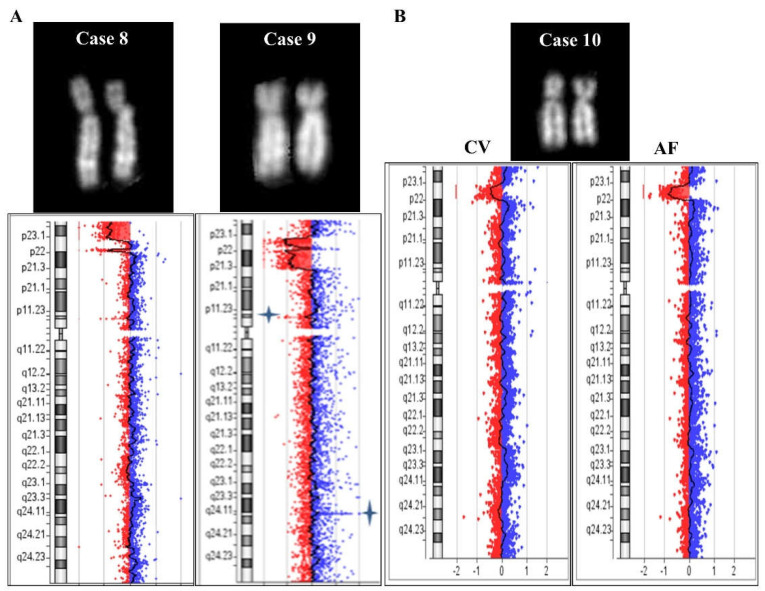
Cases 8, 9, and 10. (**A**) QFQ-banded chromosome 8 for cases 8 (left) and 9 (right), with the corresponding array CGH ideograms below. (**B**) QFQ-banded chromosome 8 of case 10 obtained from spontaneous metaphases of cytotrophoblasts. Array CGH results on chorionic villi (CV, left) and amniotic fluid (AF, right). No conventional cytogenetics were performed on amniotic fluid.

Case 8: karyotype required in a 1-month-old girl for cardiopathy and diaphragmatic hernia. The conventional karyotype from blood sampling evidenced an apparent deletion of chromosome 8p: 46,XX,del(8)(p23.1). The molecular karyotype confirmed the deletion and showed an additional centromeric deletion: arr[GRCh37] 8p23.3p23.1(191530_7752586)x1 dn,8p23.1(11536598_12404062)x1 dn (Figure 5). The conventional and molecular karyotypes of the parents were normal and signals of FISH with specific subtelomeric probes of chromosome 8 were correctly located. Owing to the availability of about 59,000 SNP probes on the microarray 4 × 180 K + SNPs used for this analysis, we found 40 informative SNPs in correspondence with the first telomeric deleted region, of which 18 were maternal and 22 were paternal. It is possible that there was a complex post-zygotic rearrangement between the two homologs. The evaluation of 28 SNPs in the disomic region between the deleted tracts allowed us to exclude the presence of uniparental disomy (UPD). Curiously, the paternal molecular karyotype showed a loss of 810 kb at 8p23.1 (from nt 6,942,281 to nt 7,752,586), which is described as polymorphism by the Database of Genomic Variants (DGV http://dgv.tcag.ca/dgv/app/home, 22 October 2021); the breakpoint of the telomeric deletion in the daughter’s case is in line with this paternal polymorphism.

Case 9: Prenatal diagnosis required because of positive first trimester combined test. A conventional karyotype from a biopsy of chorionic villi evidenced a deletion: 46,XY,del(8)(p?). A molecular karyotype confirmed and defined the anomaly: arr[GRCh37] 8p23.1(7169490_10871128)x1 dn,8p23.1p21.3(11886188_19800211)x1 dn (Figure 5). Unfortunately, it was not possible to investigate further and we were unable to determine whether two deleted regions, interrupted by a disomic trait of ~1 Mb and originating from a complex mechanism, or two distinct deletions were present on the two homologues.

Case 10: Prenatal diagnosis required because of an increased fetal nuchal translucence. A conventional karyotype from spontaneous metaphases of the biopsy of chorionic villi showed a normal situation 46,XY (Figure 5B). A molecular karyotype evidenced a mosaic condition (48%) of a deleted 8p presenting a 5.2 Mb long interstitial deletion: arr[GRCh37] 8p23.1(7169490_12404062)x1~2. A confirmatory analysis was requested on amniocytes, and the anomaly was found to be homogeneously present and no longer in a mosaic condition (Figure 5B). The possibility of maternal contamination was excluded in both cellular samples (data not shown). This region includes OMIM genes such as *MFHAS1* (OMIM605353), *RP1L1* (OMI608581), *BLK* (OMIM191305), *GATA4* (OMIM600576), and *FDFT1* (OMIM184420). This alteration is described as uncertain or potentially pathogenetic in consulted databases (Troina Database, DECIPHER https://www.deciphergenomics.org/, ClinVar https://www.ncbi.nlm.nih.gov/clinvar/, 22 October 2021) and an 8p23.1 microdeletion syndrome is described (Orphanet, ORPHA:251071). The maternal molecular karyotype was normal, while the paternal one showed a polymorphic CNV loss of 513 kb at 8p23.1, which is described as polymorphic and benign in the consulted database (ClinVar https://www.ncbi.nlm.nih.gov/clinvar/, 22 October 2021). Curiously, the telomeric breakpoint of the fetal deletion seems to be in the same region as the paternal 8p polymorphic CNV.

**Figure 6:**
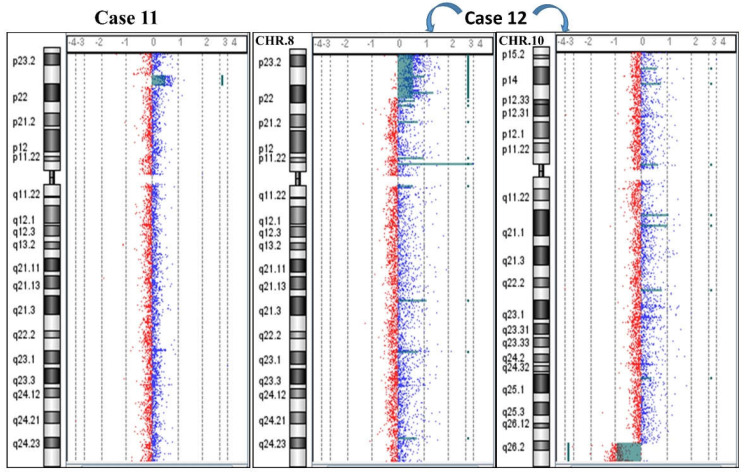
Cases 11 and 12. Array CGH results showing case 11 with a duplication (**left**) and case 12 with an 8p duplication and a 10q deletion due to an unbalanced maternal translocation t(8;10)(p22;q26.2) (**right**).

Case 11: Molecular karyotype required in a 10-year-old boy with language delay, aortic bicuspidia, and dysmorphic features. A duplication of 3.8 Mb was evidenced:

arr[GRCh37] 8p23.1(8111027_11906094)x3 dn (Figure 6). The molecular karyotypes of both parents were normal, without any apparent CNVs that might be considered mediators of the rearrangement.

Case 12: An investigation was conducted on suspicion of a familiarity with a chromosomal anomaly in two siblings with a clinical phenotype: the 3-year-old sister had language delay and dysmorphic features; the 6-year-old brother had mild psychomotor retardation and corpus callosum hypoplasia. In addition, the mother was carrier of a balanced translocation: t(8;10)(p22;q26.2). Both the children inherited the same unbalanced derivative, explaining the clinical phenotype in both cases (Figure 6).

The sister’s molecular karyotype was arr[GRCh37] 8p23.3p22(194617_16010296)x3,10q26.2q26.3(129699867_135434178)x1 mat. The brother’s molecular karyotype was arr[GRCh37] 8p23.3p22(119720_15545878)x3,10q26.2q26.3(129699867_135512075)x1 mat. The differences detected between siblings at the first breakpoint of chromosome 8p were ascribed to technical effects.

### 2.3. Benign CNVs on 8p

**Figure 7:**
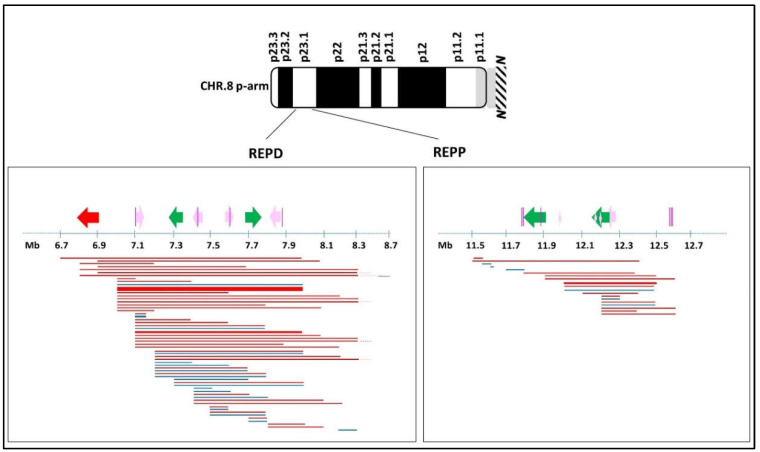
Schematic diagram of benign CNVs on 8p identified in our cohort. Defensin gene clusters and OR/FAM clusters are represented by colored arrows: red, α-defensin; green, β-defensin; pink, OR/FAM clusters. The direction of the arrows indicates cluster orientation. Purple vertical lines: OR7 family at 8p23.1. Horizontal lines: benign CNVs: losses are red; gains are blue. Line thickness: frequency of the CNV in our cohort where the thinnest is for ≤40 patients; the largest is for the most frequent CNV (≥500 patients); the middle lines are for intermediate situations (around 100 patients). The region of the 8q arm not shown in the figure is indicated by the N.

A total of 3633 consecutive molecular karyotypes (from 2013 to 2020) were revised from the two laboratories to search for benign CNVs on 8p. We found 2489 benign CNVs: 551 in gain and 1938 in loss. Interestingly, 75.5% of the total mapped onto one of the following recurrent breakpoints: the majority of these (1414, i.e., 57% of the total) falls between nt 6,754,549 and nt 8,768,319, near REPD, of which only 241 (17%) are gain (medium length 398kb) and 1173 (83%) are loss (medium length 812kb). A minority of benign CNVs (466, i.e., 19% of the total) falls between nt 11,477,642 and nt 12,742,082, near REPP, of which only 21 (4.5%) are gain (medium length 159kb) and 445 (95.5%) are loss (medium length 432 kb) (Figure 7).

## 3. Discussion

Deletions and duplications of the terminal region of 8p have been studied using cytogenetics for a long time. In 1992, Hutchinson et al. described the cases of five patients with deletion 8p23 and another three patients recovered from the literature, similar in phenotype and karyotype, and previously described as 8p- (without indication of involved band region). In the discussion, they assumed that such deletions could occur through a recombinational mechanism between sister chromatids, or intrachromosomally, facilitated by repeat sequences near the telomere and within the chromosome [10]. Subsequently, this hypothesis was confirmed by others [1,2] and, finally, Lupski and co-workers detailed the mechanism, naming it nonallelic homologous recombination (NAHR) [11,12].

Recent studies on the region 8p23.1 have confirmed the presence of highly polymorphic regions in correspondence with the olfactory receptor gene clusters or the defensin repeat (ORDRs), identifying them as highly unstable and dynamic structures, as previously stated [13,14]. The 12 cases we described in this study demonstrate the fragility of 8p. Figure 1 shows how all the breakpoints identified in this study seem to be arranged without solution of continuity from the telomere through almost the entire length of the short arm (see Figure S1 for a detailed view by the UCSC Genome Browser of deleted and duplicated regions). Overall, it is possible to identify three main clusters where breakpoints occur (Figure 1): one telomeric and two more proximal, all responsible for mechanisms that lead to chromosomal aberrations due mostly to meiotic errors triggered by highly polymorphic and repetitive sequences. These clusters can be considered responsible for repetitive mechanisms of meiotic errors which, on the one hand, constitute the engine of variability and genomic evolution but, on the other, cause human pathological rearrangements [15,16].

In the last decades, several studies have reported cases with isolated microdeletions and duplications of 8p23 [17,18,19,20,21]. Most of these aberrations are interstitial, but the terminal region appears to be compromised in inv dup rearrangements. In cases 8, 9, 10, and 11, an NAHR could be hypothesized with a single (cases 10 and 11) or double (cases 8 and 9) recombination event (Figure 5, Figure 6 and Figure S2A,B).

The mechanism producing the inv dup del(8p) was initially proposed by Floridia et al. in 1996, and is now universally accepted [1]. These rearrangements are recurrently mediated by the two olfactory receptor gene clusters or defensin repeat (ORDRs). In addition, the polymorphic 8p23 inversion between these clusters increases the susceptibility of 8p to rearrangements [2,3,7]. Cases 1–7 may have undergone this mechanism. In particular, an NAHR between these sequences during the meiotic pairing can result in the formation of an intermediate dicentric chromosome and an acentric fragment, which is inevitably lost. The dicentric chromosome then undergoes a random break between the two centromeres during the first anaphase, leading to the possible formation of a chromosome containing duplicate sequences interrupted by a disomic region of variable extension. This event may have occurred in cases 1, 2, 3, and 7 (Figure 1, Figure 4 and Figure S2C). The mechanism may also include the phenomenon of telomere capture (Figure S2D). In case 7, we ascertained this through FISH analysis: the capture of telomere 8q probably stabilized the 8p end generated after the random break of the intermediate dicentric chromosome. This phenomenon has been described numerous times in the case of 8p [22,23,24,25] and for other chromosomes [23,26,27,28]. However, other mechanisms that stabilize the extremity generated by the breaking of the dicentric intermediate might include the formation of a new telomeric cap by ‘telomere healing’, in which telomeric sequences can be acquired de novo [29] or the stabilization that occurs through circularization of the inv dup del chromosome, leading to the formation of a ring [30,31]. Conversely, for cases 4, 5, and 6, the most likely mechanism seems to involve an initial double-strand break of the two sister chromatids. Fusion of the broken ends results in a symmetric U-type reunion between the sister chromatids, leading to the formation of a dicentric chromosome. Breakage distal to the fusion site outside the fusion region results in a monocentric chromosome with a terminal deletion and an inverted duplication without a single copy region between the duplication (Figure S2E).

Finally, it is important to remember that the presence of numerous families of OR genes distributed throughout the human genome may facilitate and promote meiotic errors through recombination of homologous sequences between nonhomologous chromosomes, generating reciprocal translocations. This is the case of the recurrent chromosomal translocation t(4;8)(p16;p23) and probably for the t(8;10)(p22;q26.2) described in case 12. Curiously, the FAM7, an OR subfamily, seems to be located close to the breakpoints identified in the two siblings through the molecular karyotype on 8p23.3 and 10q26.3 [9]. In this case, the two siblings inherited both a partial duplication on 8p and a partial deletion on 10q due to segregation adjacent to 1 after meiotic pairing of the maternal t(8;10)(p22;q26.2) (Figure 6 and Figure S2F,G).

In summary, we reported 12 unpublished cases of 8p aberrations using molecular karyotype. The picture we paint, owing also to cases reviewed in the literature, is that of a continuum of fragility on chromosome 8p, specifically at 8p23.1, due to the particular presence of homologous sequences both intrachromosomal and interchromosomal. It is necessary to pay attention to these sequences due to the risk of formation of new chromosomal aberrations with pathological effects.

## 4. Materials and Methods

### 4.1. Participants

Informed consent was collected for all patients. The study was conducted following the normal diagnostic procedures of the Clinical Pathology Department, S. Gerardo Hospital, Monza, Italy [32]. Participants were enrolled after written informed consent was obtained from parents or legal guardians.

### 4.2. Chromosome Analysis

The cases were collected from two medical genetics laboratories that applied the standard methods to conduct the chromosome analysis, following the Italian Society of Human Genetics (SIGU) guidelines [33] and subsequent modifications online (https://sigu.net/category/linee-guida-e-raccomandazioni/, last access 22 October 2021). In summary, the PHA-stimulated lymphocyte cultures were collected after 72 h and the slides were stained with QFQ or GTG (only for case 7) banding. Amniocytes were cultured using standard techniques, and chromosomal preparations were performed both in suspension and in situ. Spontaneous metaphases from cytotrophoblast of chorionic villus samples were analyzed using standard techniques. Karyotypes were expressed following the guidelines of the International System for Cytogenomic Nomenclature 2020 (ISCN 2020) [34]. All images were captured at 100× magnification. Some of them were then enlarged photographically.

### 4.3. FISH Analysis

Fluorescence in situ hybridization (FISH) was carried out according to the manufacturer’s protocol. In particular, a ToTelVysion Multi-color FISH Probe kit (Vysis, Abbott Park, IL, USA) was used.

The BAC probes used in the FISH study were selected according to the University of California, Santa Cruz (UCSC) Genome Browser (GRCh37/hg19, release February 2009). BAC probes were generated through nick translation and then labelled with biotin-dUTP (Deoxyuridine triphosphate) (Roche, Basel, Switzerland), digoxigenin-Dutp (Roche, Basel, Switzerland), or Cy3-dUTP (Amersham Biosciences, Little Chalfont, UK).

All digital images were captured using a Leitz microscope (LeicaDMRA2 or LeicaDM5000B, Leica Microsystems GmbH, Leica Microsystems, Milan, Italy), equipped with a charge coupled device (CCD) camera (Leica Microsystems, Milan, Italy) and analyzed by means of various software (Leica CW4000 or Chromowin). All images were captured at 100× magnification. Some of them were then enlarged photographically.

### 4.4. Array Comparative Genomic Hybridization (Array CGH)

Genomic DNA was purified from peripheral blood mononucleate cells using a GenElute™Blood Genomic DNA kit (Sigma-Aldrich, Darmstadt, Germany) or a Wizard Genomic DNA Purification Kit (PromegaTM, Mannheim, Germany), according to the supplier’s instructions. Genomic DNA from amniotic fluid samples and chorionic villi biopsy was obtained using an iPrep nucleic acid purification system (Thermo Fisher Scientific, Waltham, MA, USA) and then purified using a DNA Clean&Concentrator^®^ kit (Zymo Research Corporation, Irvine, CA, USA). DNA concentration was determined on a NanoDrop ND-1000 spectrophotometer (NanoDrop Technologies, Berlin, Germany) and Qubit fluorometer (Thermo Fisher Scientific, Waltham, MA, USA). Array comparative genomic hybridization analysis was performed using a SurePrint G3 Human CGH Microarray kit 8 × 60 K (cases 2, 3, 5 and 6) or a Human Genome CGH Microarray kit 4 × 44 K (case 1) with a mean resolution of ~130 kb (Agilent Technologies, Palo Alto, CA, USA). For all other cases, array CGH analysis was performed using an Agilent SurePrint G3 Human CGH plus SNP Microarray 4 × 180 K (Agilent Technologies, Palo Alto, CA, USA), following the manufacturer’s instructions. DNA control reference was provided by Agilent Technologies. The array was scanned at 2 or 3 µm resolution using an Agilent microarray scanner and analyzed using Agilent Cytogenomics software v3.0.6.6 and v5.0 (Agilent Technologies, Palo Alto, CA, USA). Significant chromosomal aberration was determined using the algorithm ADM-2 (threshold, 5.0; absolute minimum average log2 ratio, 0.20; with at least three or more consecutive probe sets; see more details in [32]). Nucleotide designations were made according to the GRCh37/hg19 build of the human genome. In case of placenta biopsy and amniotic fluid cells, maternal contamination was excluded using a PowerPlex^®^ 16 HS System kit (Promega Corporation, Madison, WI, USA).

### 4.5. UCSC Genome Browser Analysis

Selected regions of the 8p arm were mapped using the UCSC genome browser hg19 assembly (https://genome.ucsc.edu/cgi-bin/hgGateway, 22 October 2021). The mapping of OR/FAM loci was performed according to Glusman [9].

## Data Availability

Data available on request.

## Supplementary Materials

The following are available online at https://www.mdpi.com/article/10.3390/ijms23063347/s1.
