# Supplementary material for: Characterization of Chromosomal Breakpoints in 12 Cases with 8p Rearrangements Defines a Continuum of Fragility of the Region
Source: Int J Mol Sci. 2022 Mar 20;23(6):3347. doi: 10.3390/ijms23063347 (PMC8954119; doi:10.3390/ijms23063347)
Supplement: Supplementary file 1 [file ijms-23-03347-s001.zip › Fig S1.pdf]

# Case 1: deletion

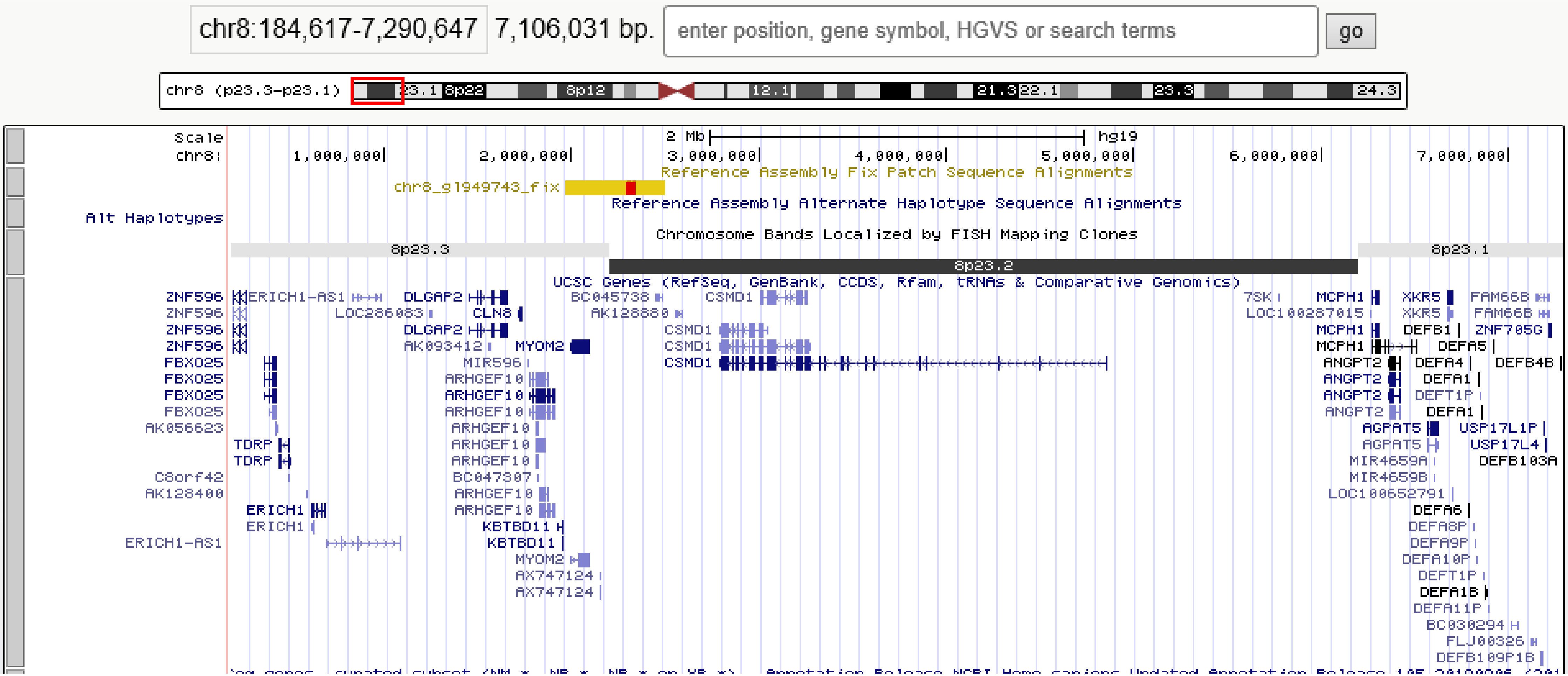

# Case 1: duplication

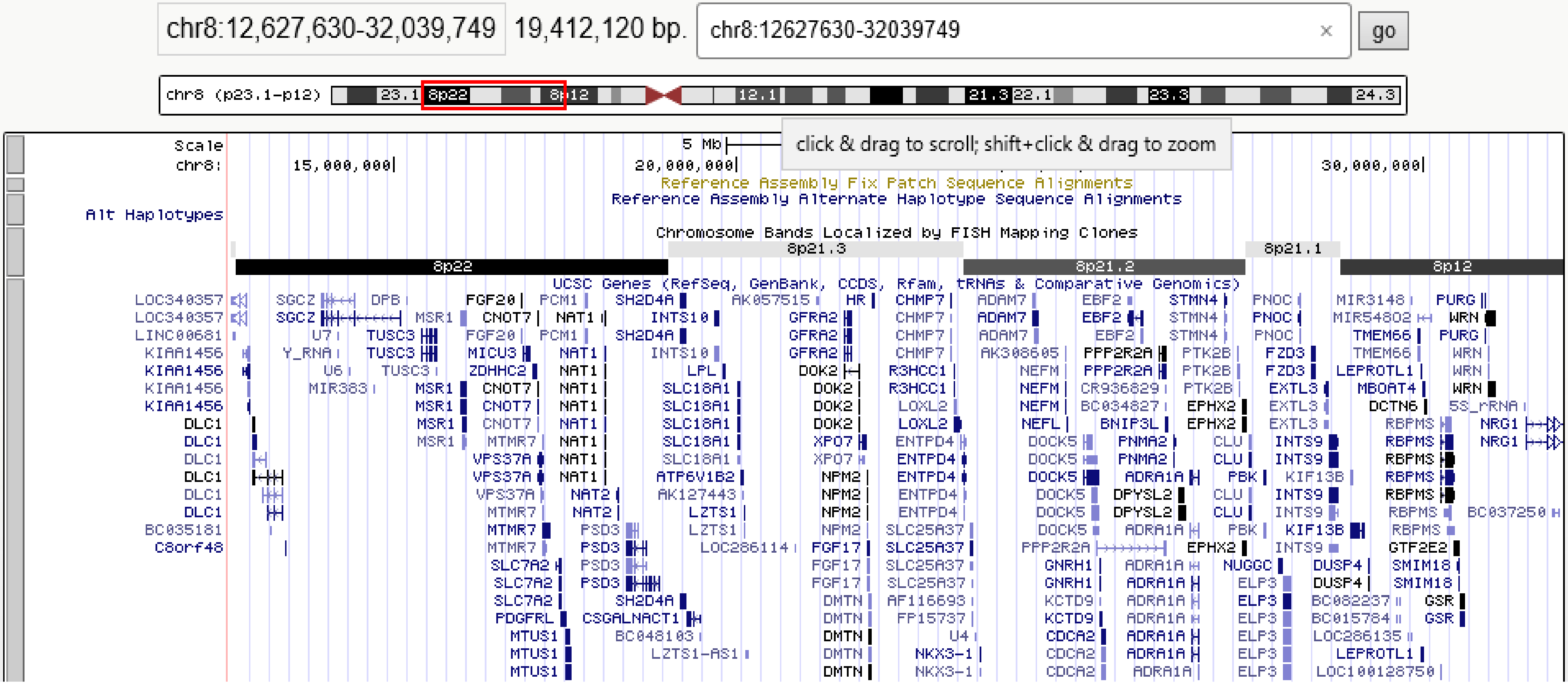

# Case 2: deletion

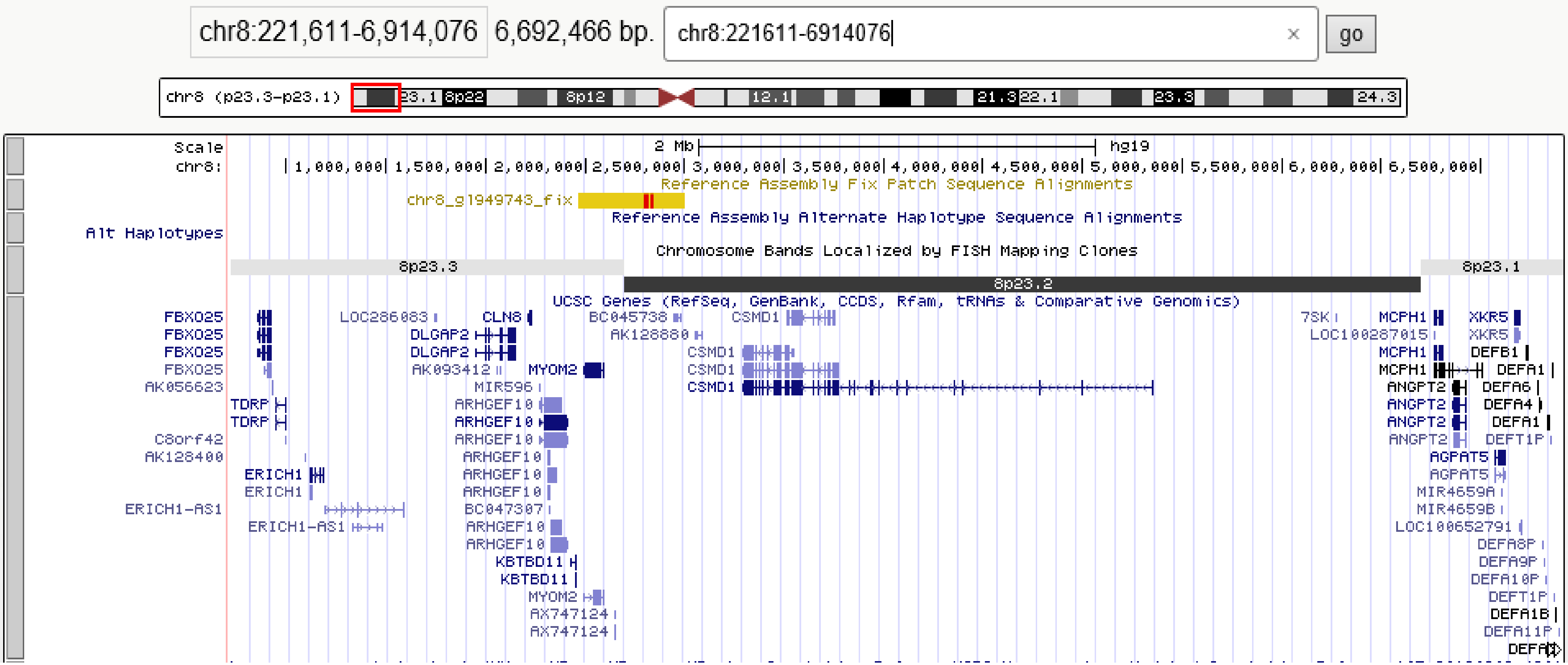

# Case 2: duplication

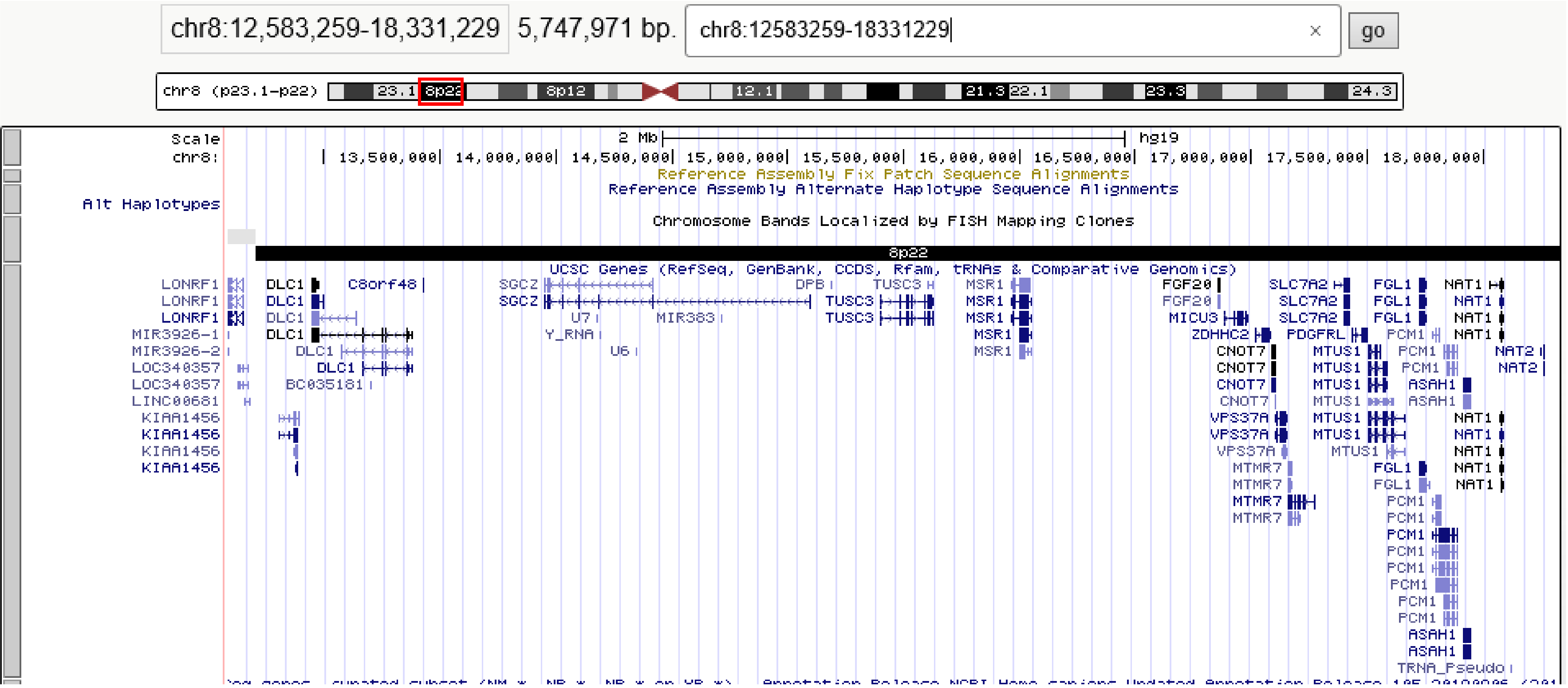

# Case 3: deletion

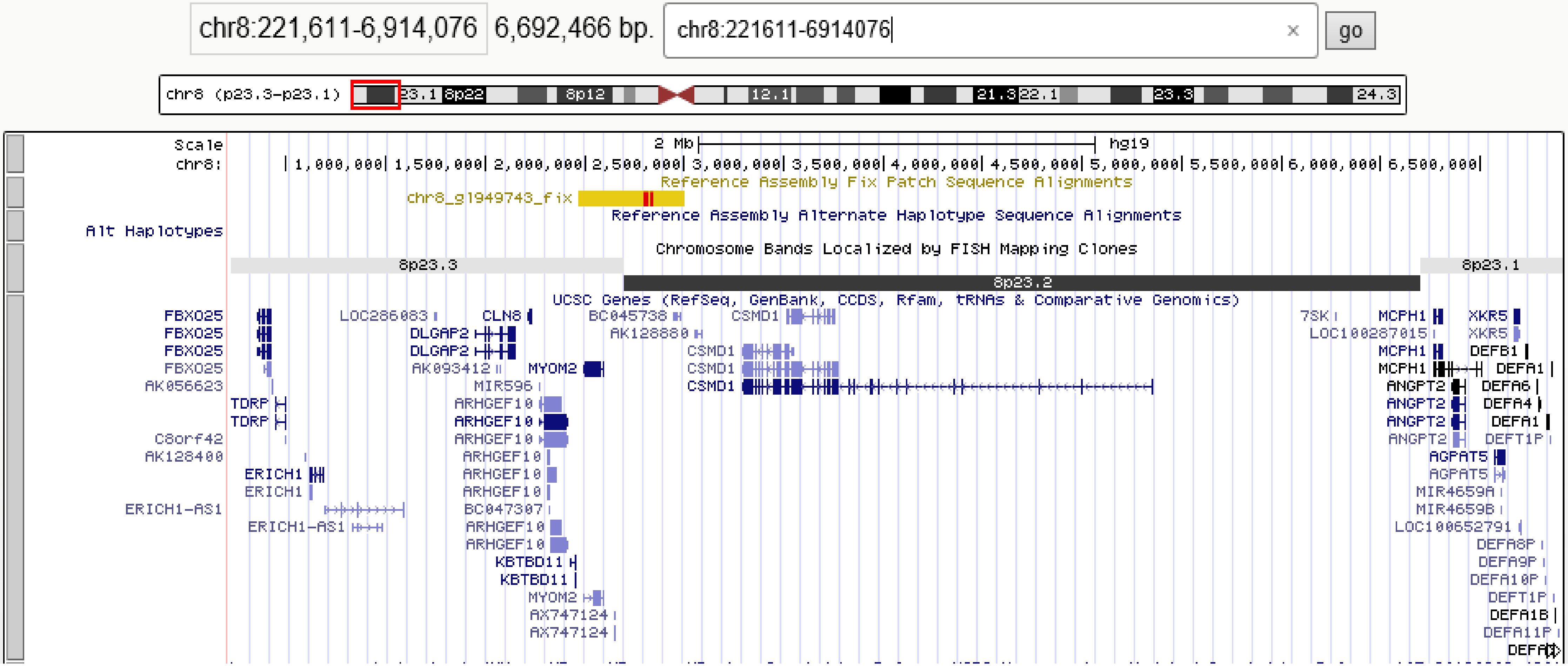

# Case 3: duplication

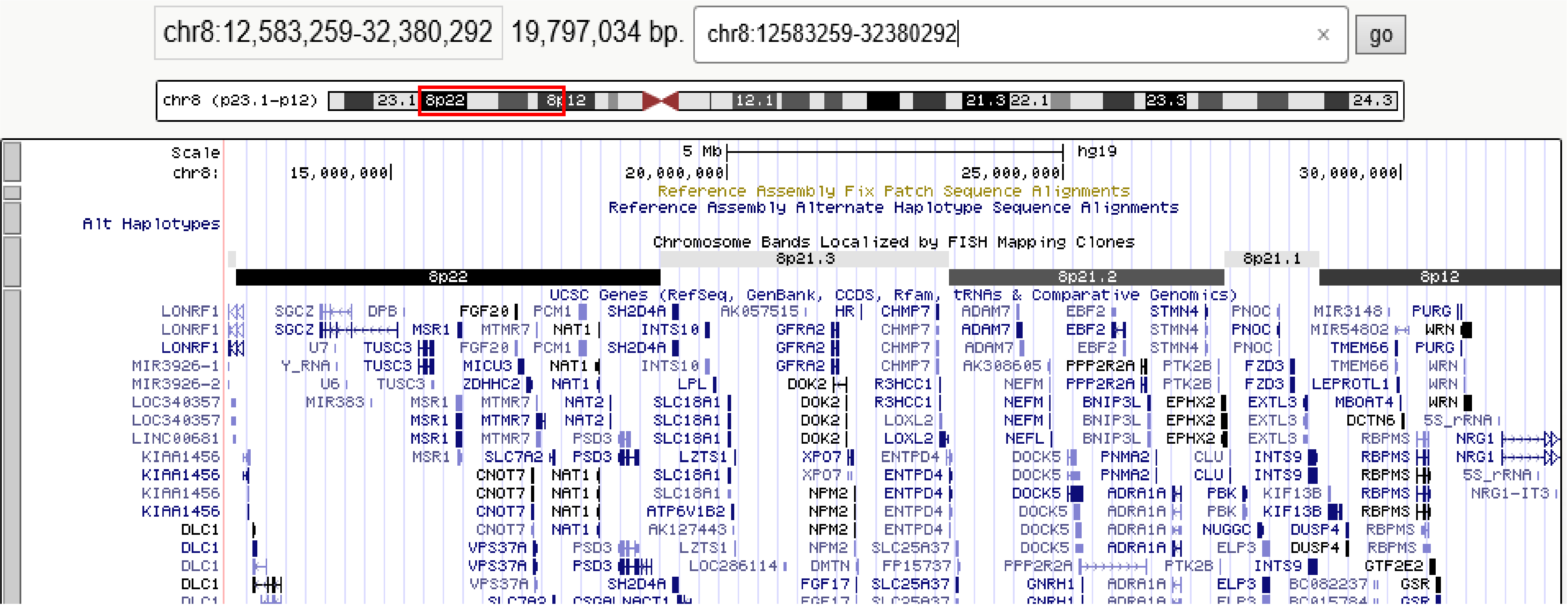

# Case 4: deletion

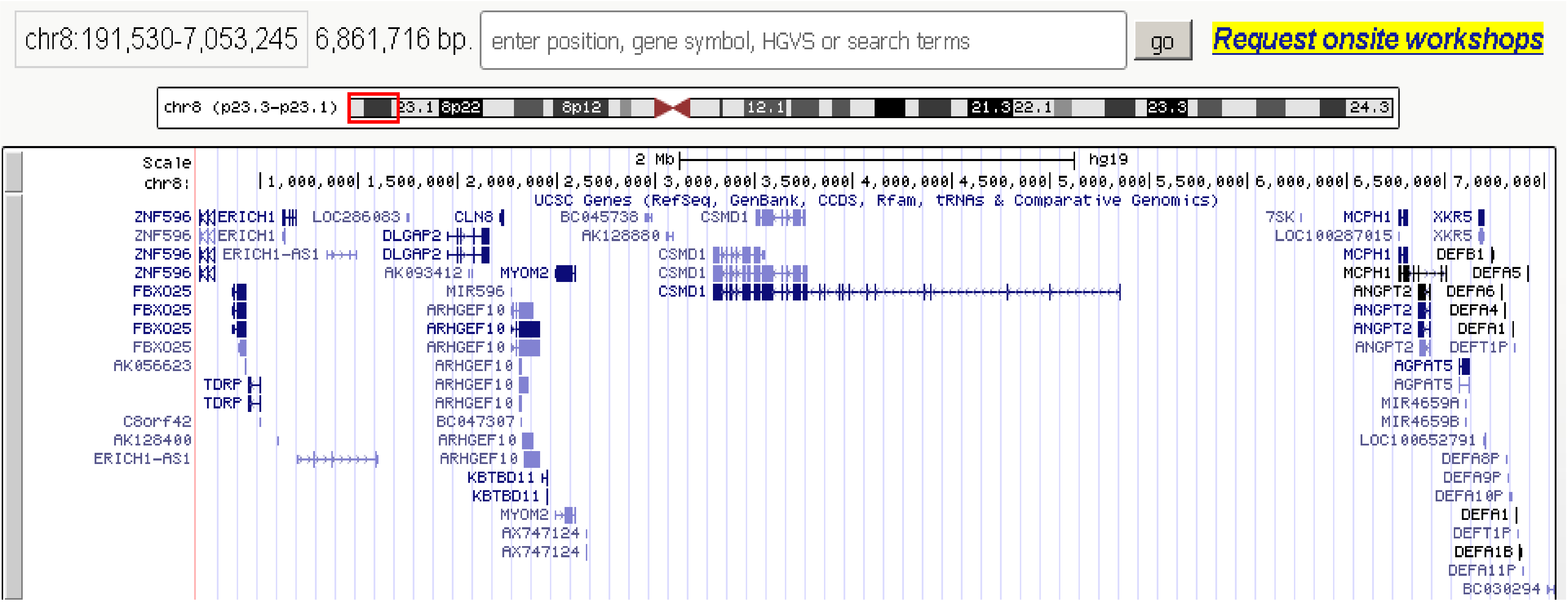

# Case 4: duplication

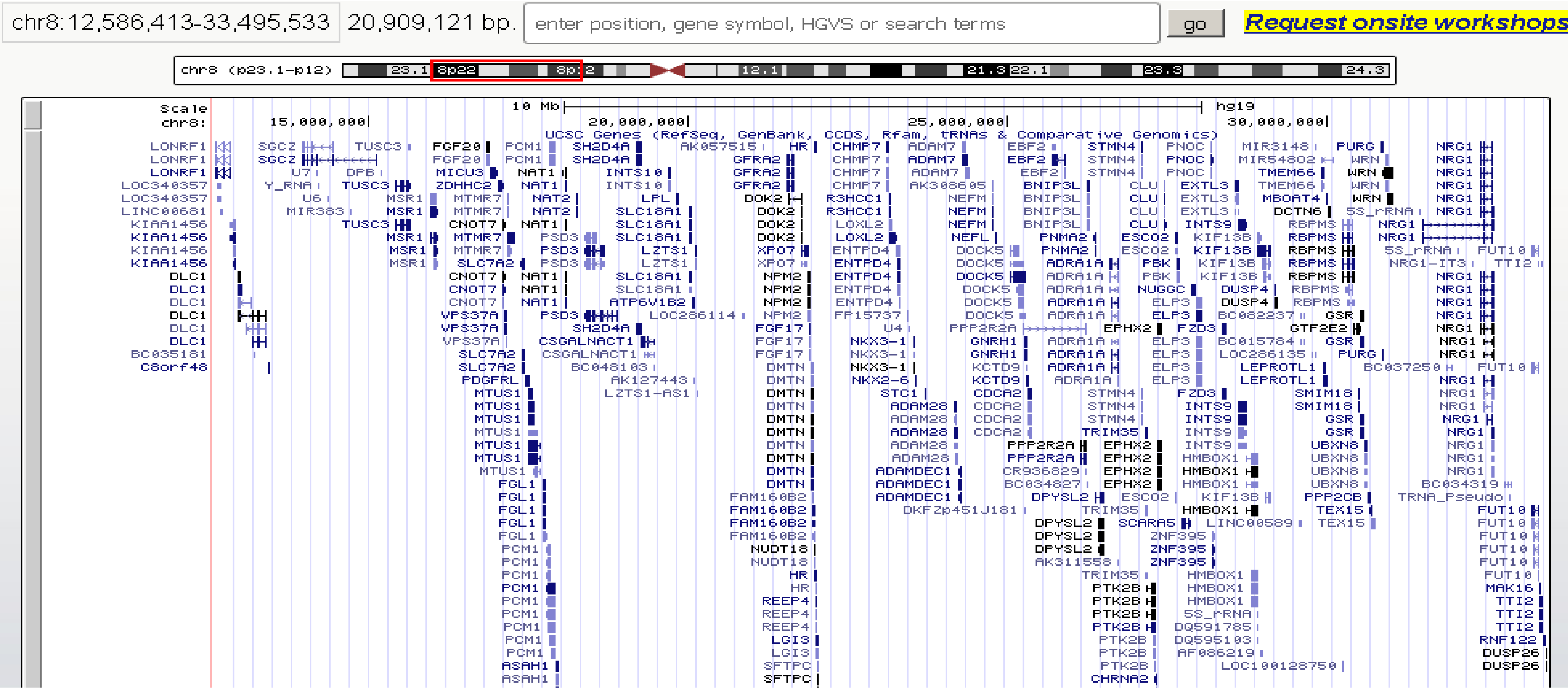

# Case 5: deletion

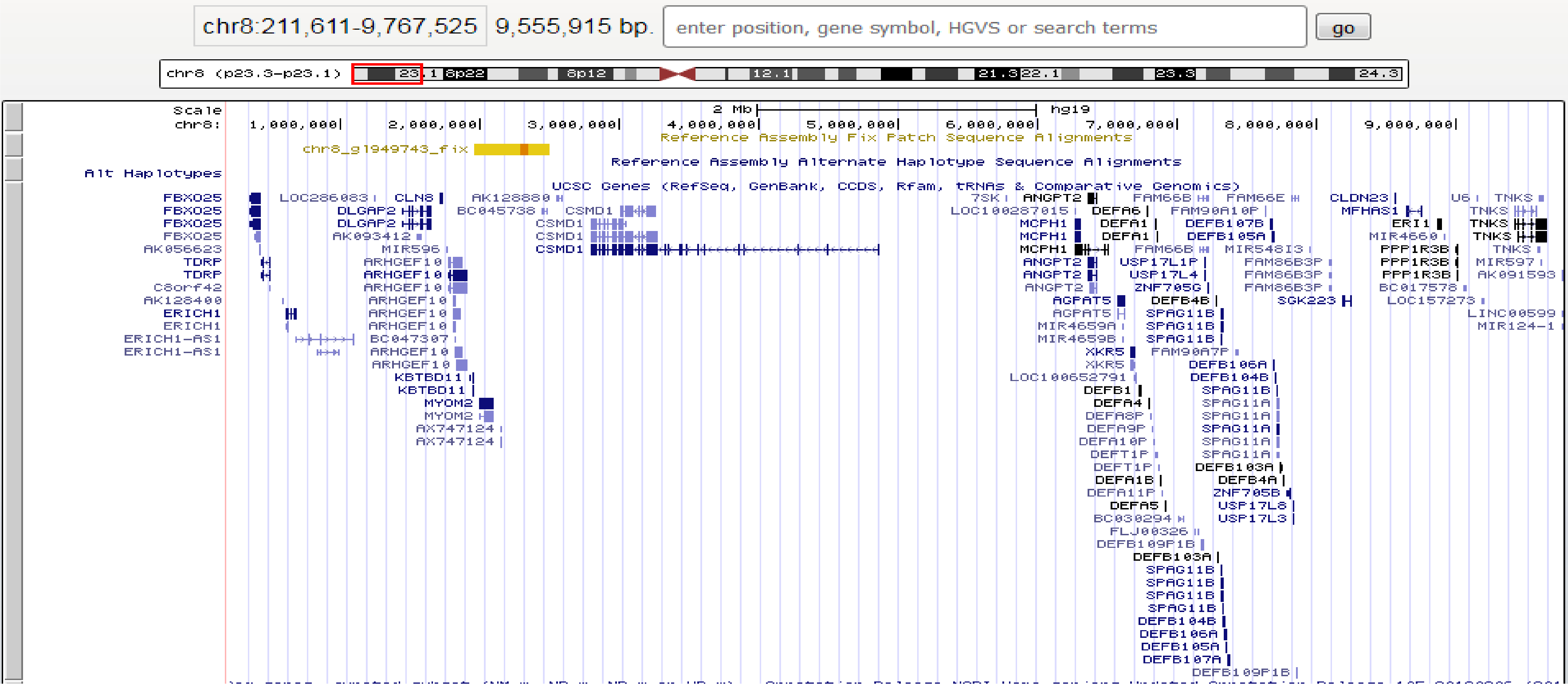

# Case 5: duplication

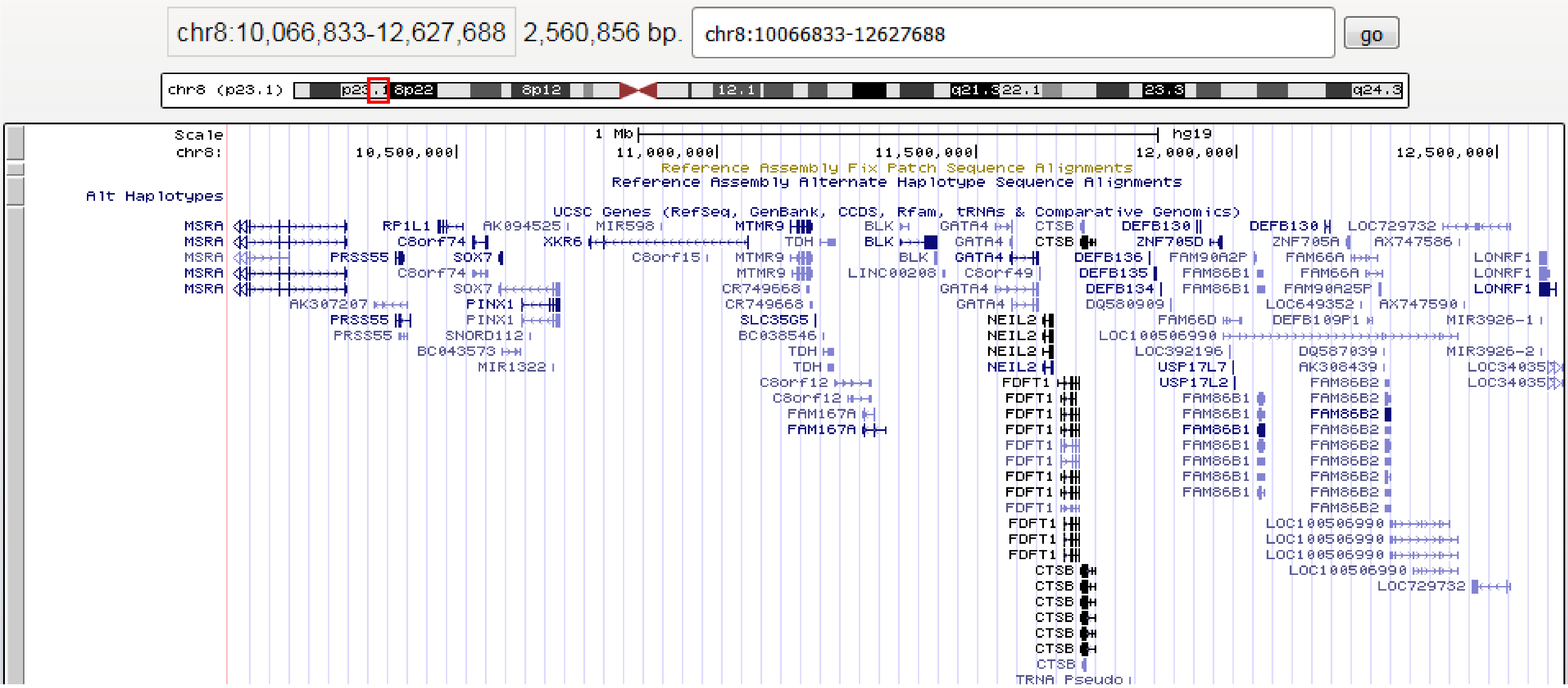

# Case 6: deletion

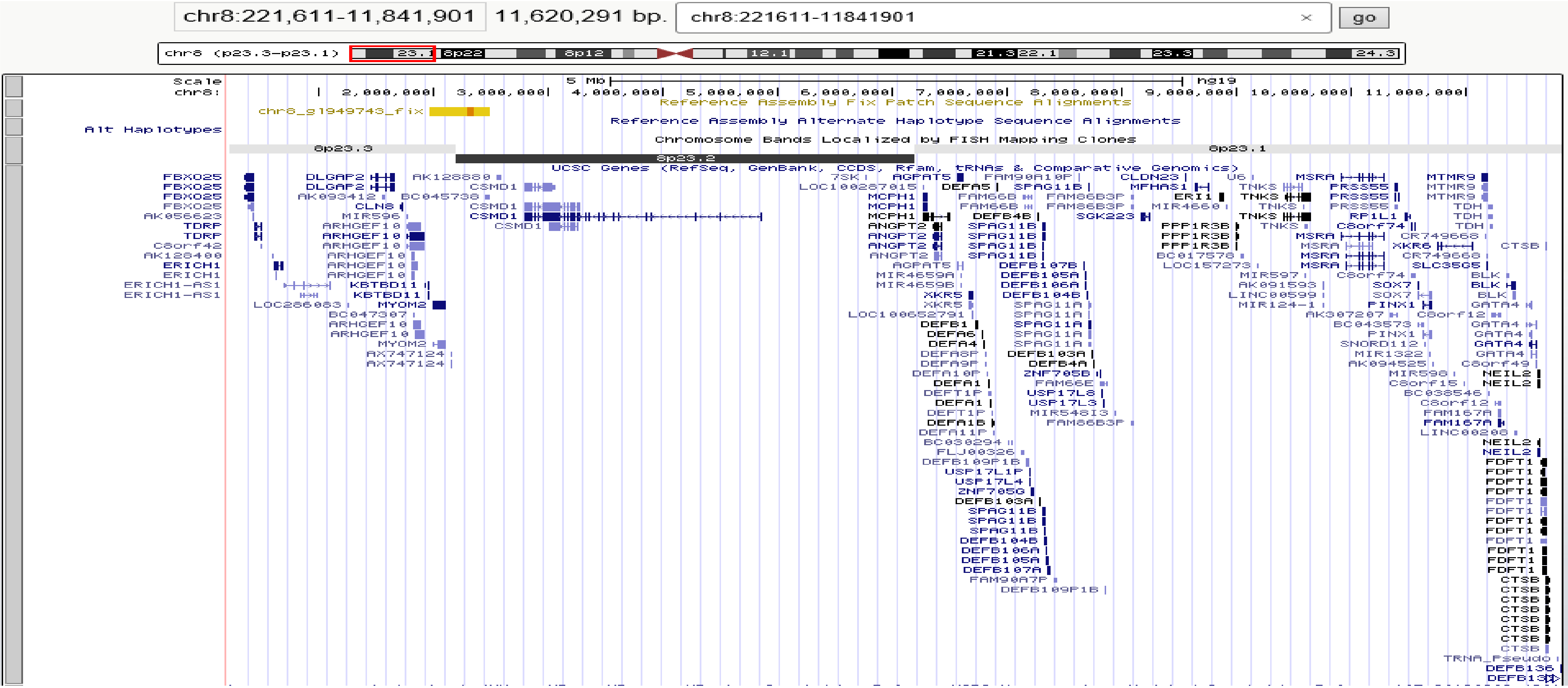

# Case 6: duplication

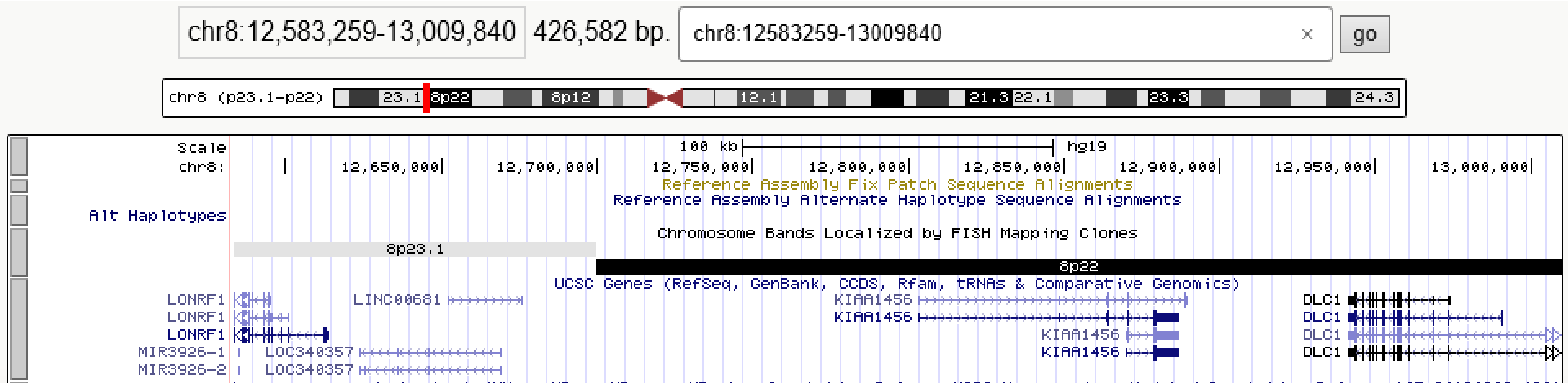

# Case 7: deletion

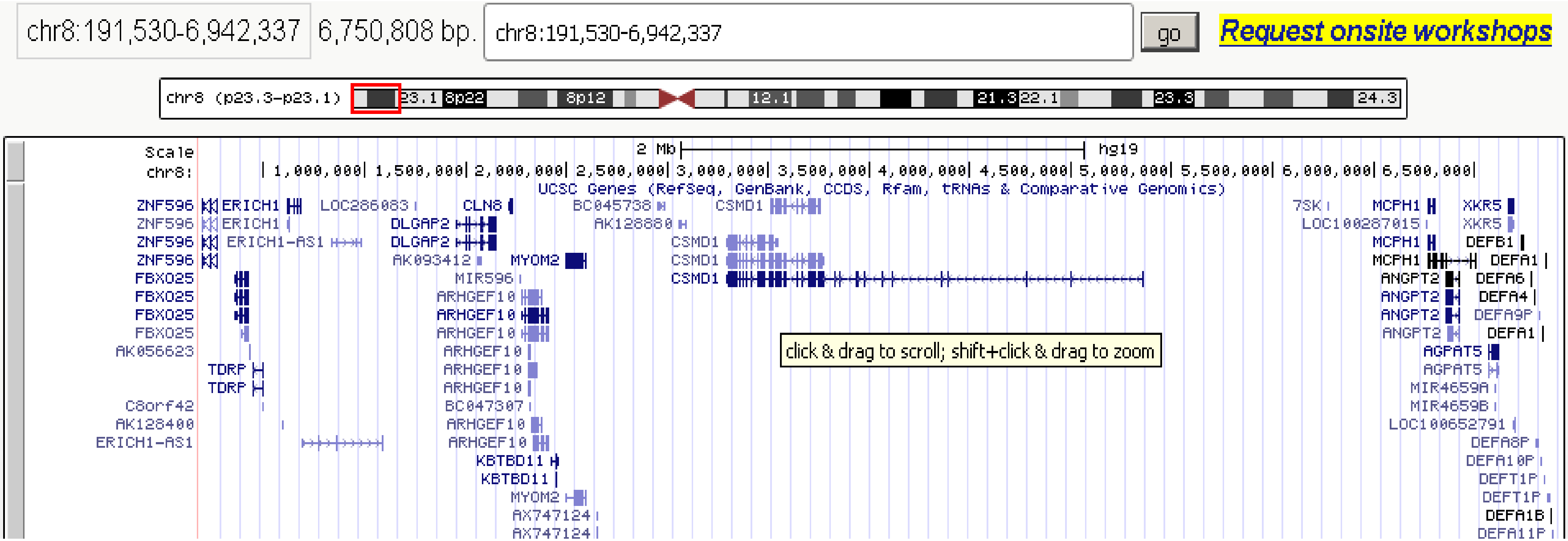

# Case 7: duplication

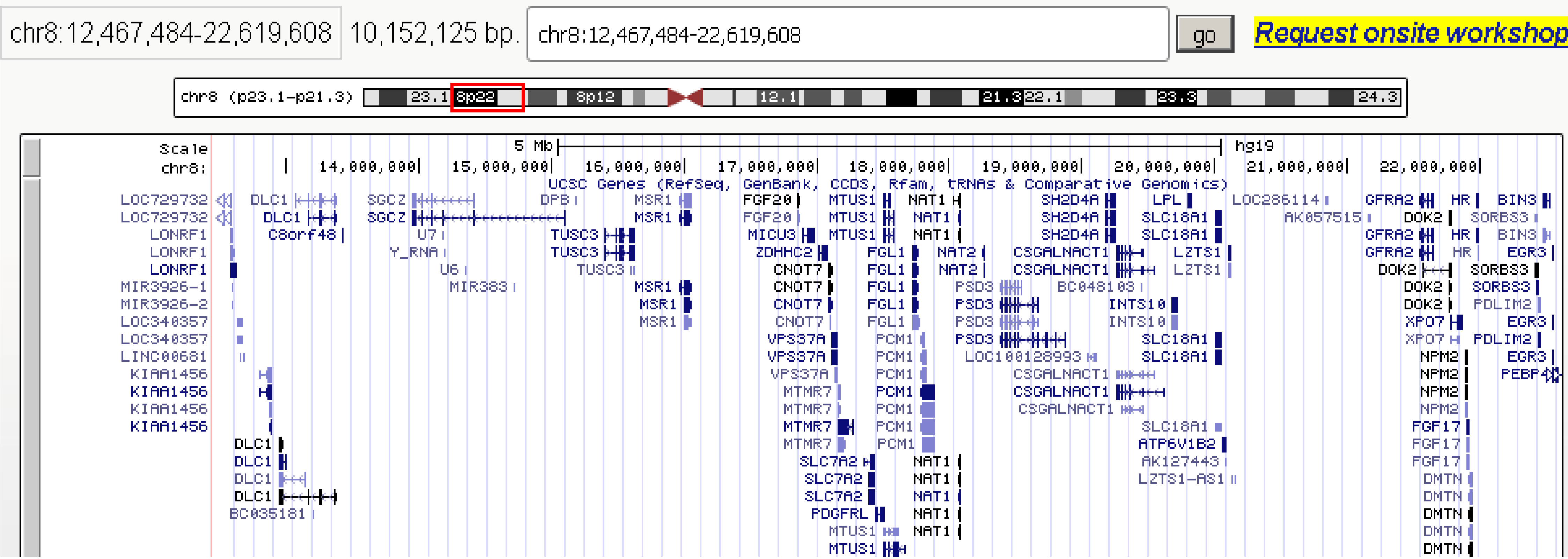

# Case 8: first deletion

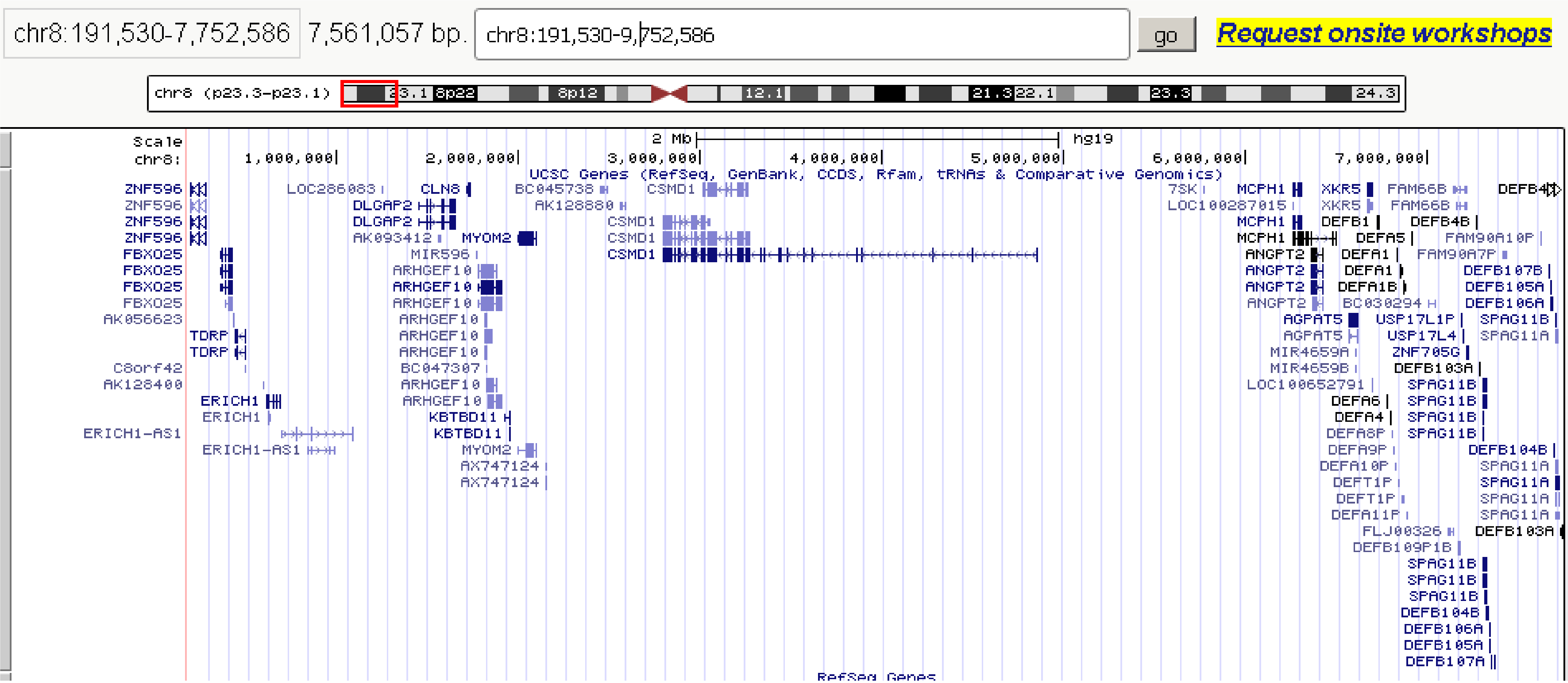

# Case 8: second deletion

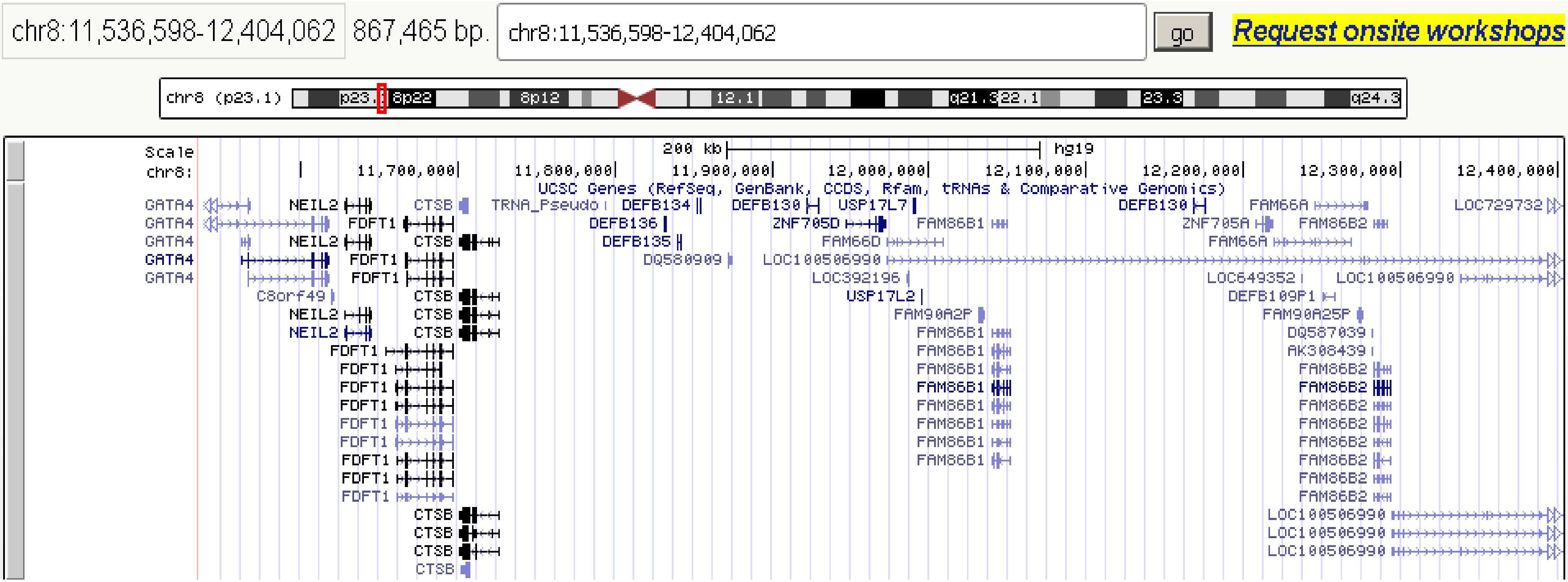

# Case 9: first deletion

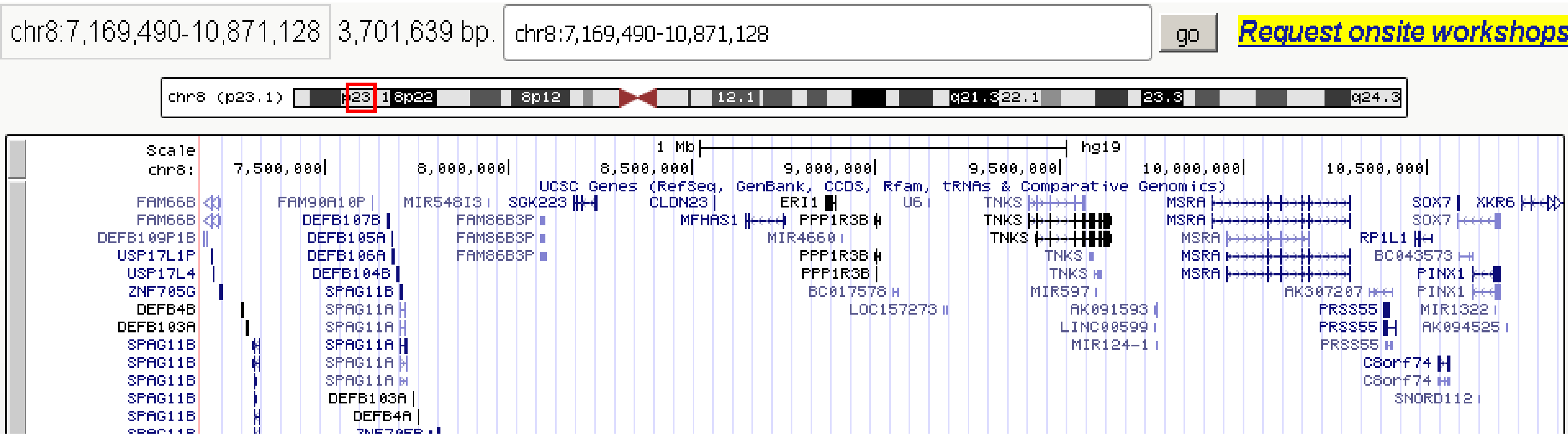

# Case 9: second deletion

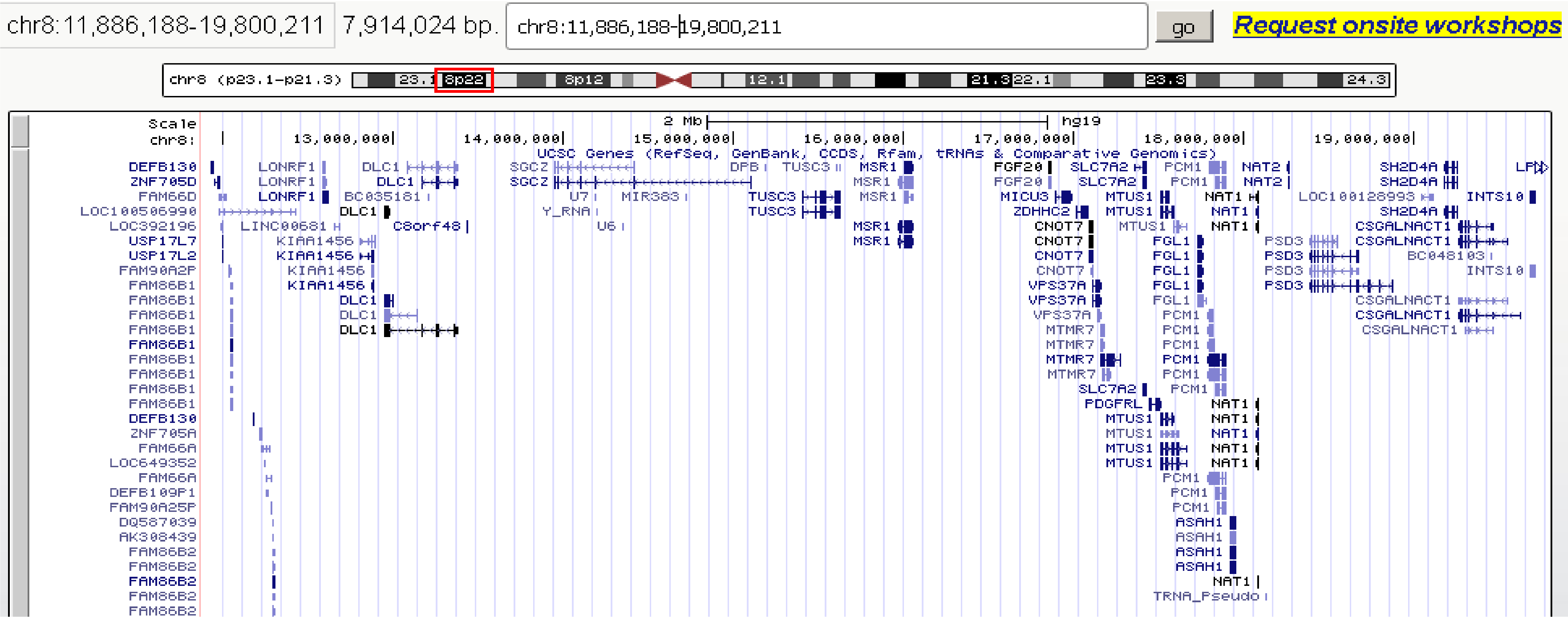

# Case 10 : deletion (AF)

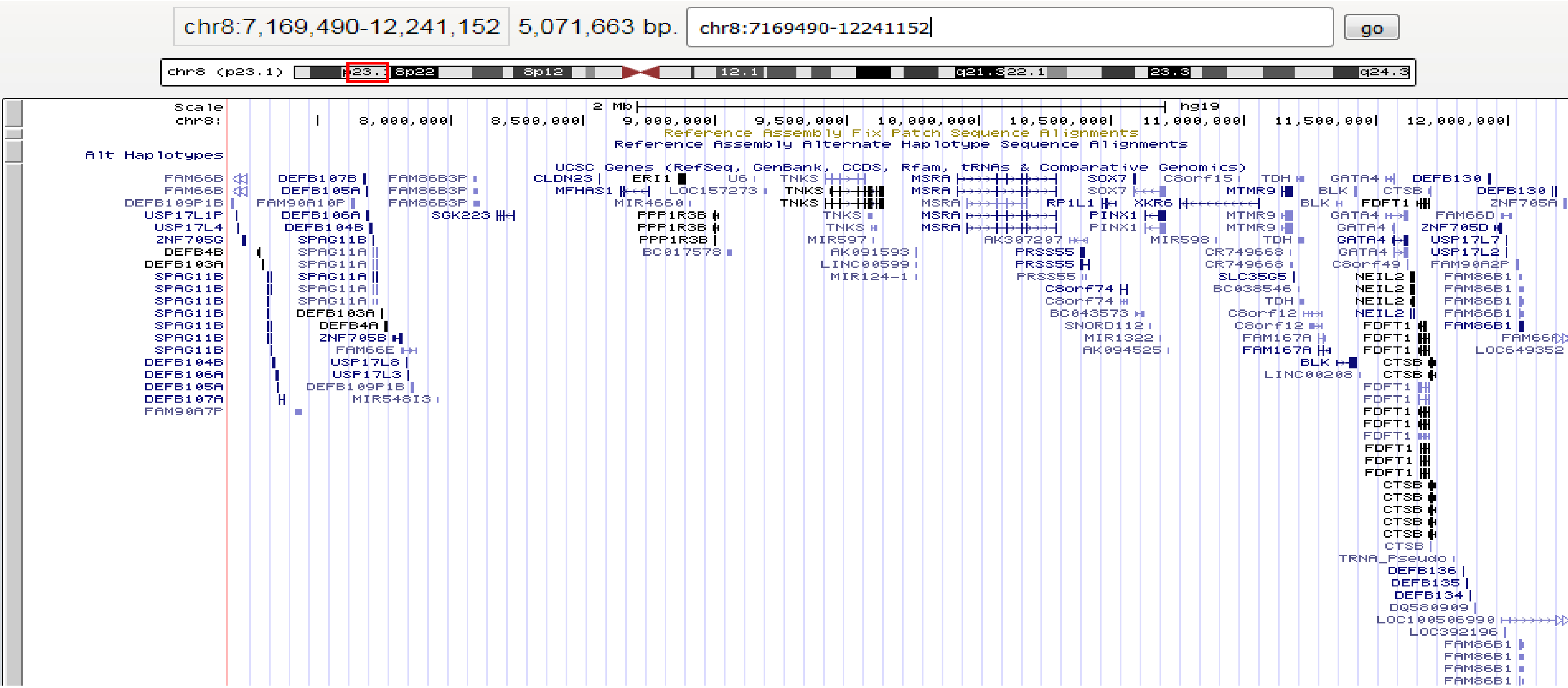

# Case 11

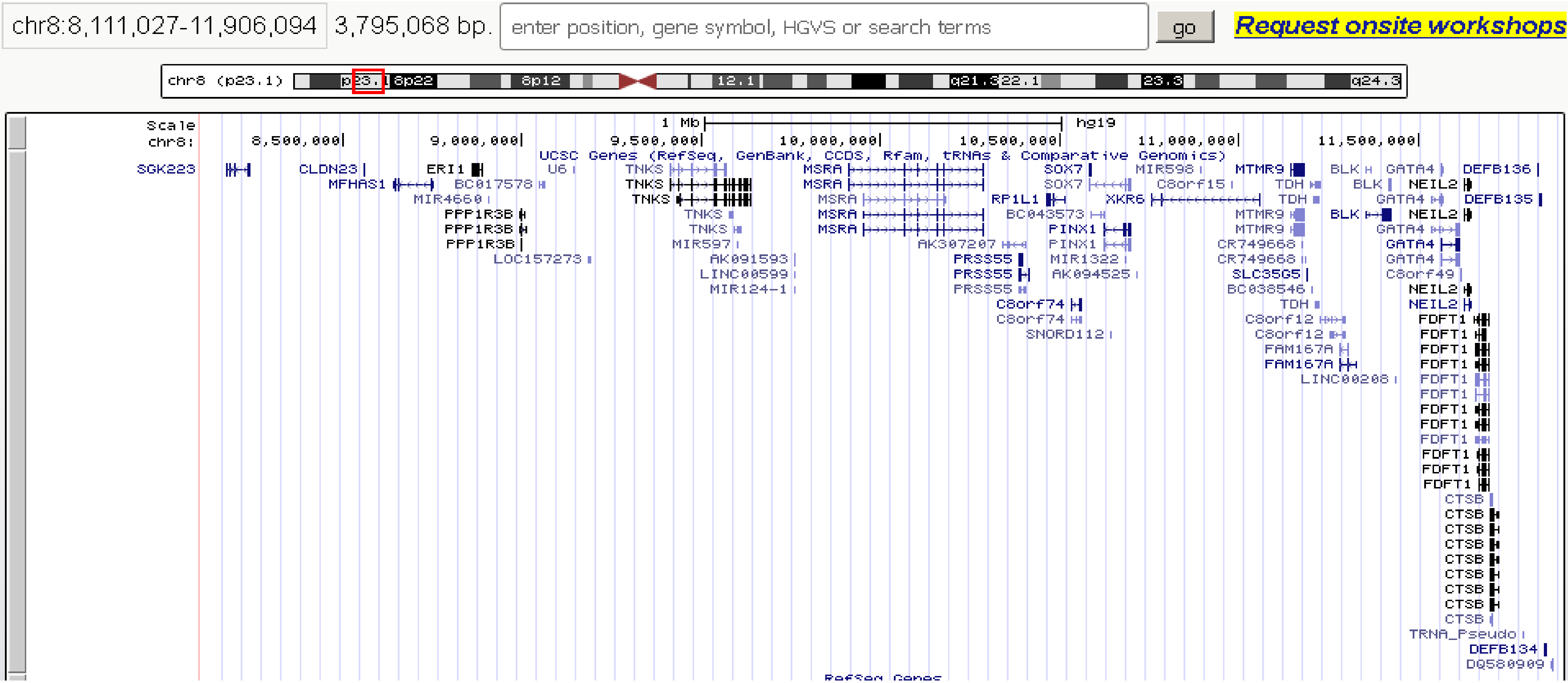

chr8:8,111,027-11,906,094
3,795,068 bp.
enter position, gene symbol, HGVS or search terms
go
Request onsite workshops
chr8 (p23.1)
p23.1
8p22
8p12
12.1
q21.3
22.1
23.3
q24.3
Scale
chr8:
1 Mb
hg19
8,500,000
9,000,000
9,500,000
10,000,000
10,500,000
11,000,000
11,500,000
UCSC Genes (RefSeq, GenBank, CCDS, Rfam, tRNAs & Comparative Genomics)
SGK223
CLDN23
MFHAS1
ERI1
BC017578
MIR4660
PPP1R3B
LOC157273
U6
TNKS
TNKS
TNKS
TNKS
TNKS
MIR597
AK091593
LINC00599
MIR124-1
MSRA
MSRA
MSRA
MSRA
MSRA
AK307207
RP1L1
BC043573
PRSS55
PRSS55
PRSS55
C8orf74
C8orf74
SNORD112
SOX7
SOX7
XKR6
PINX1
PINX1
MIR1322
AK094525
MIR598
C8orf15
MTMR9
TDH
TDH
MTMR9
MTMR9
CR749668
CR749668
SLC35G5
BC038546
TDH
C8orf12
C8orf12
FAM167A
FAM167A
LINC00208
BLK
BLK
BLK
GATA4
GATA4
GATA4
GATA4
GATA4
C8orf49
NEIL2
NEIL2
NEIL2
NEIL2
FDFT1
CTSB
TRNA_Pseudo
DEFB136
DEFB135
DEFB134
DQ580909
RefSeq Genes

# Case 12

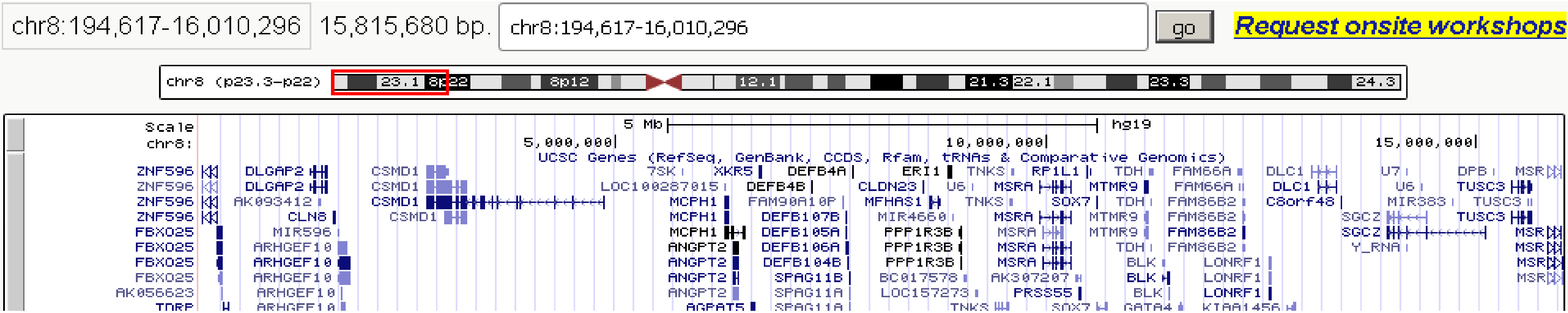
chr8:194,617-16,010,296
15,815,680 bp.
chr8:194,617-16,010,296
go
Request onsite workshops
chr8 (p23.3-p22)
UCSC Genes (RefSeq, GenBank, CCDS, Rfam, tRNAs & Comparative Genomics)
hg19

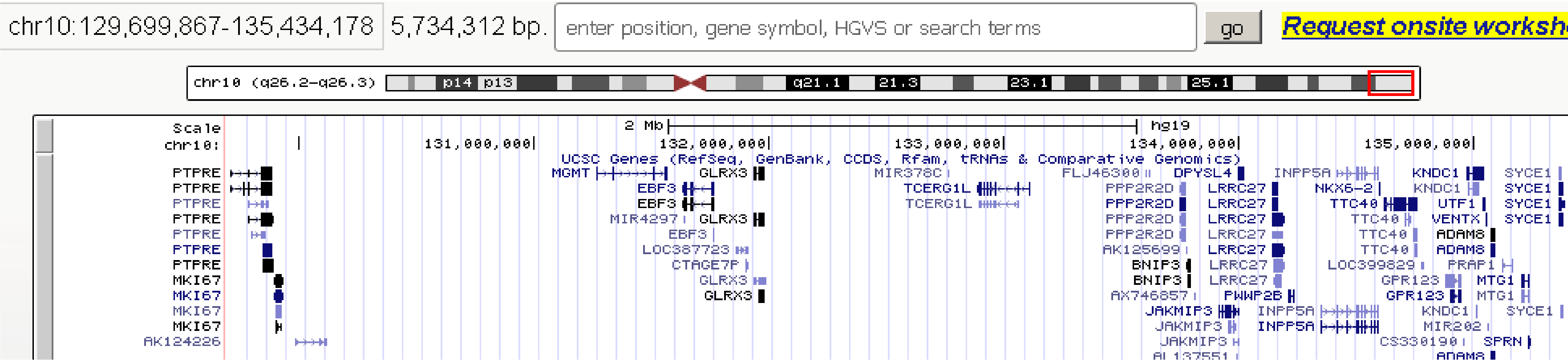
chr10:129,699,867-135,434,178
5,734,312 bp.
enter position, gene symbol, HGVS or search terms
go
chr10 (q26.2-q26.3)
UCSC Genes (RefSeq, GenBank, CCDS, Rfam, tRNAs & Comparative Genomics)
hg19
