# Supplementary material for: Characterization of Chromosomal Breakpoints in 12 Cases with 8p Rearrangements Defines a Continuum of Fragility of the Region
Source: Int J Mol Sci. 2022 Mar 20;23(6):3347. doi: 10.3390/ijms23063347 (PMC8954119; doi:10.3390/ijms23063347)
Supplement: Supplementary file 1 [file ijms-23-03347-s001.zip › Fig S2 mod.pdf]

A

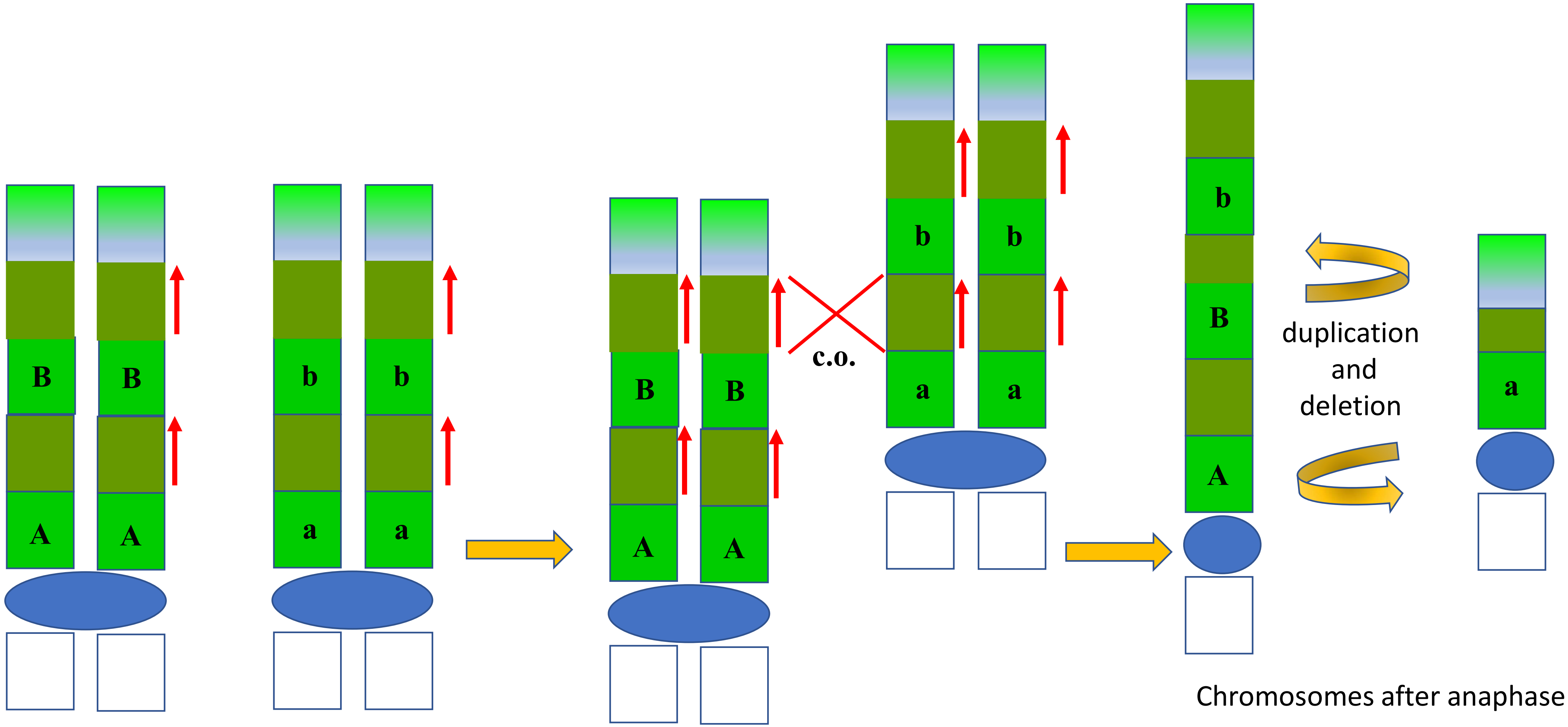
B
B
A
A
b
b
a
a
B
B
A
A
c.o.
b
b
a
a
b
B
A
duplication
and
deletion
a
Chromosomes after anaphase

B

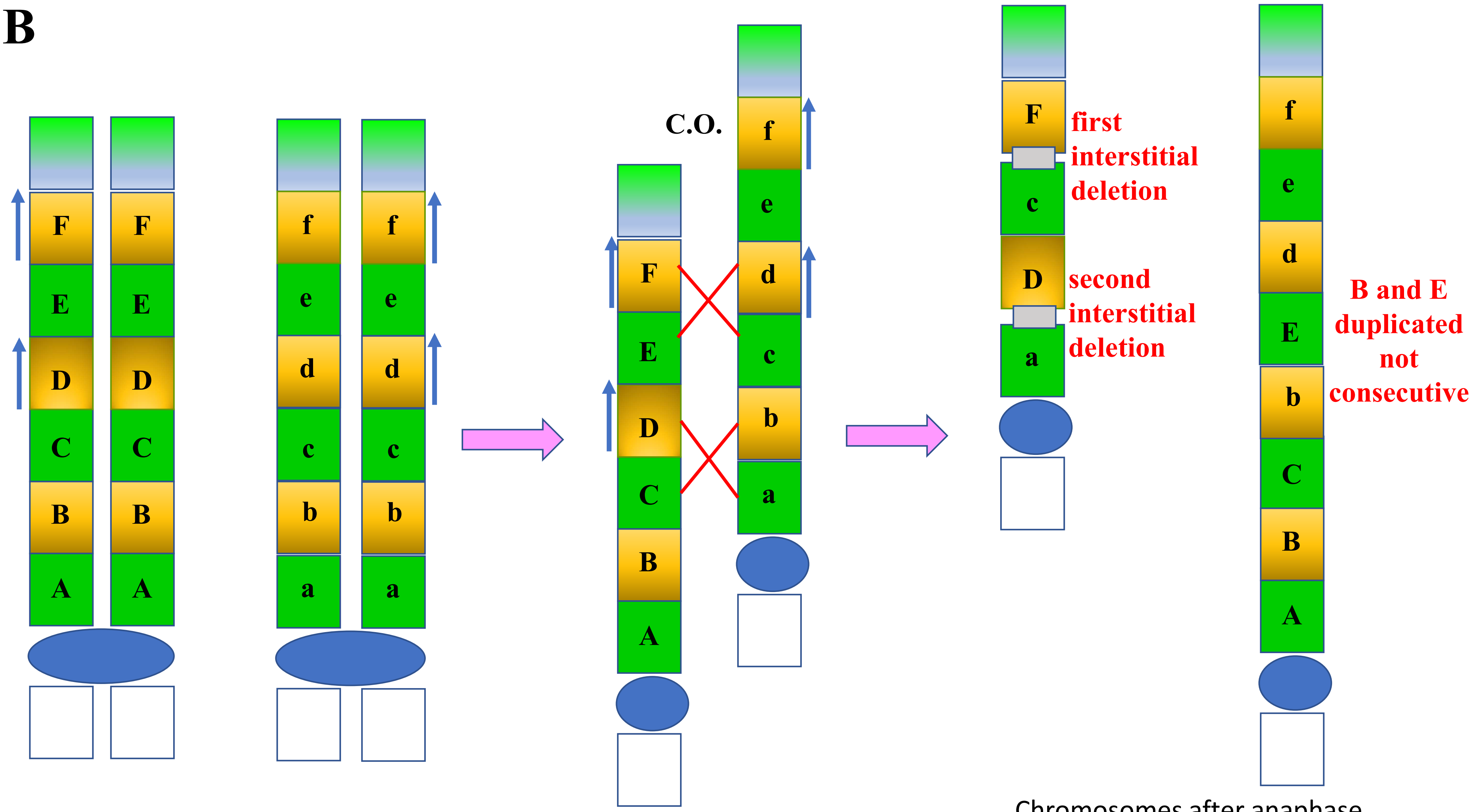

Chromosomes after anaphase

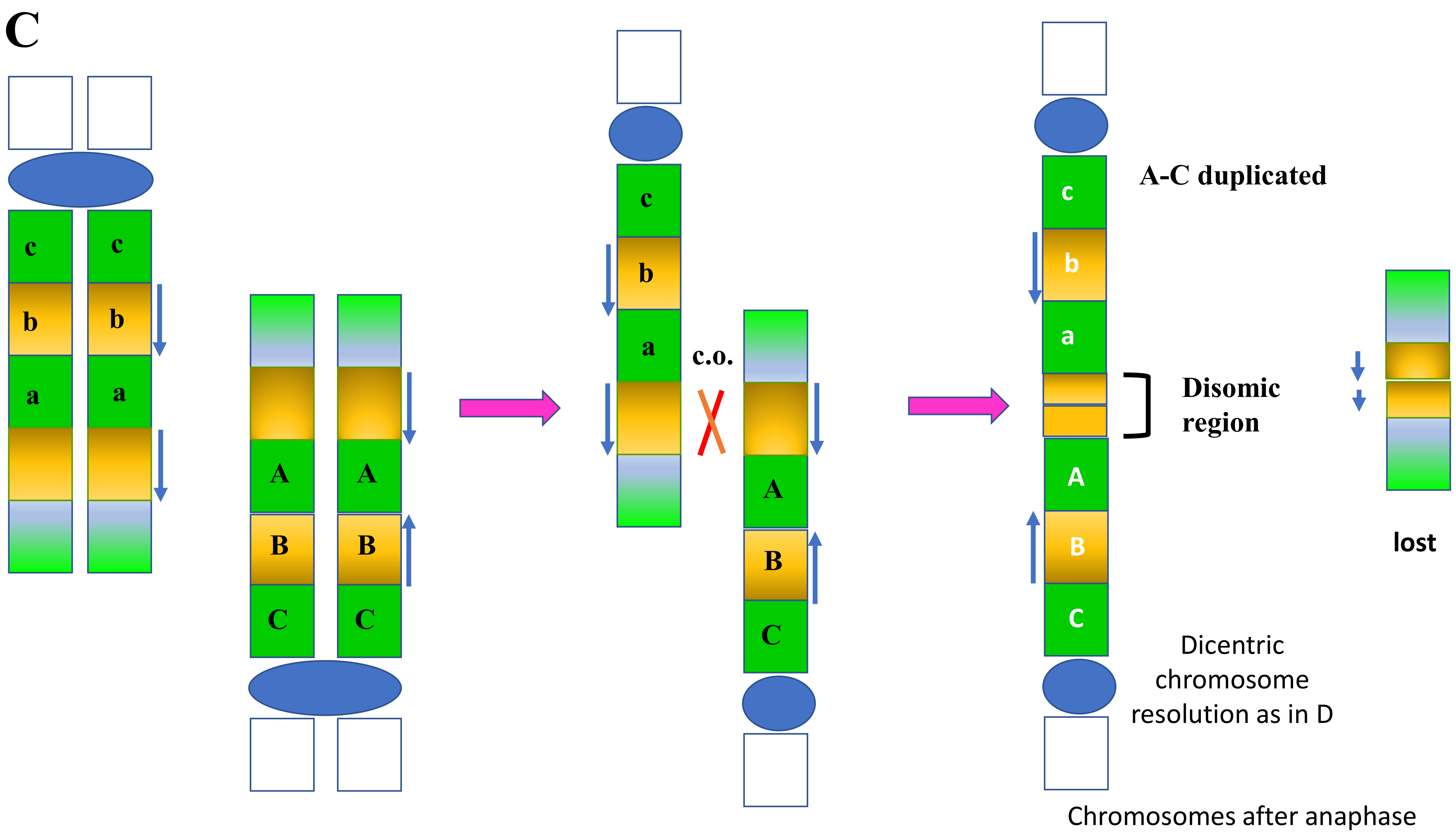
C
c
b
a
c
b
a
A
B
C
A
B
C
c
b
a
c.o.
A
B
C
c
b
a
A-C duplicated
Disomic
region
A
B
C
lost
Dicentric
chromosome
resolution as in D
Chromosomes after anaphase

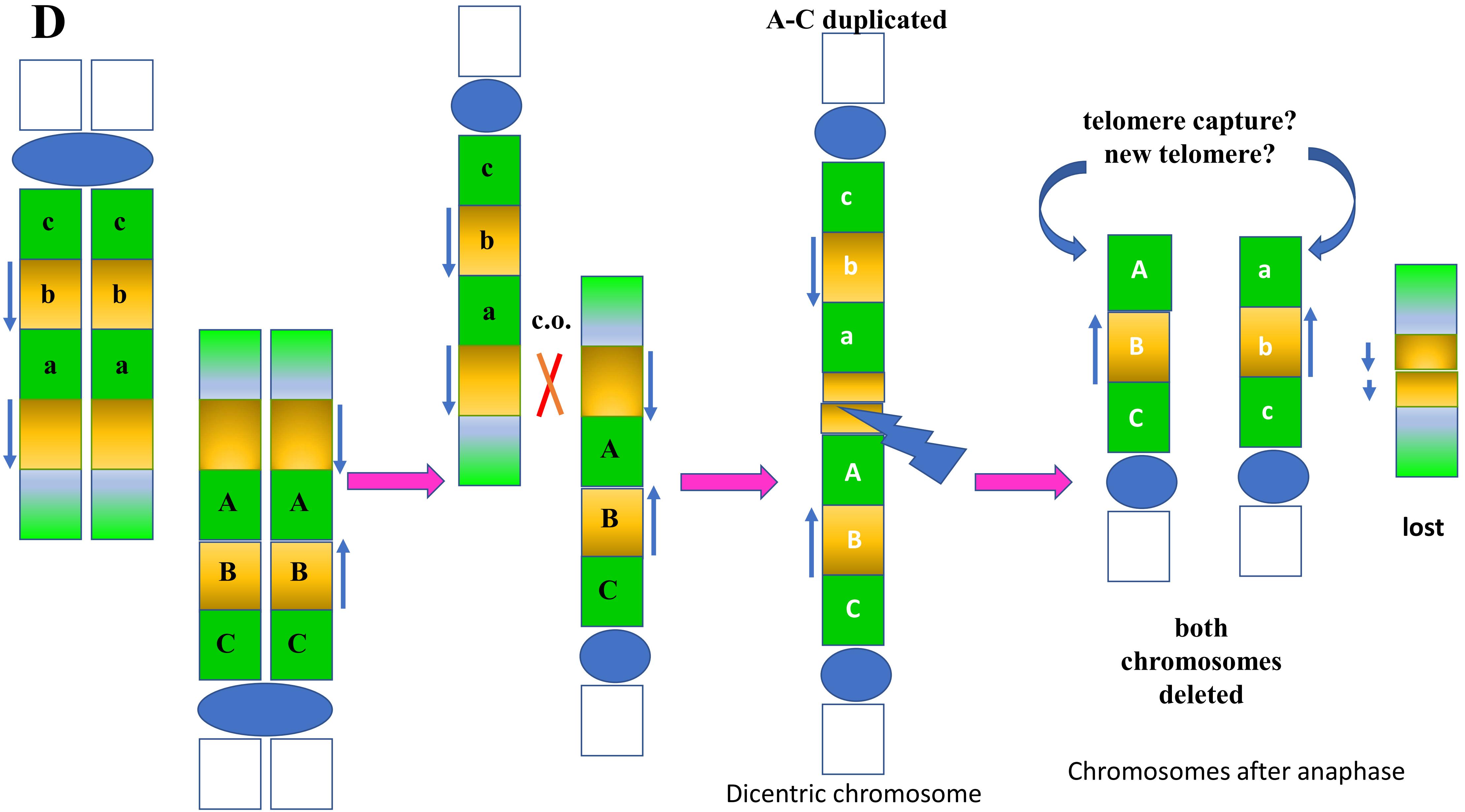
D
c
b
a
c
b
a
A
B
C
A
B
C
c
b
a
c.o.
A
B
C
A-C duplicated
c
b
a
A
B
C
Dicentric chromosome
telomere capture?
new telomere?
A
B
C
a
b
c
lost
both
chromosomes
deleted
Chromosomes after anaphase

E

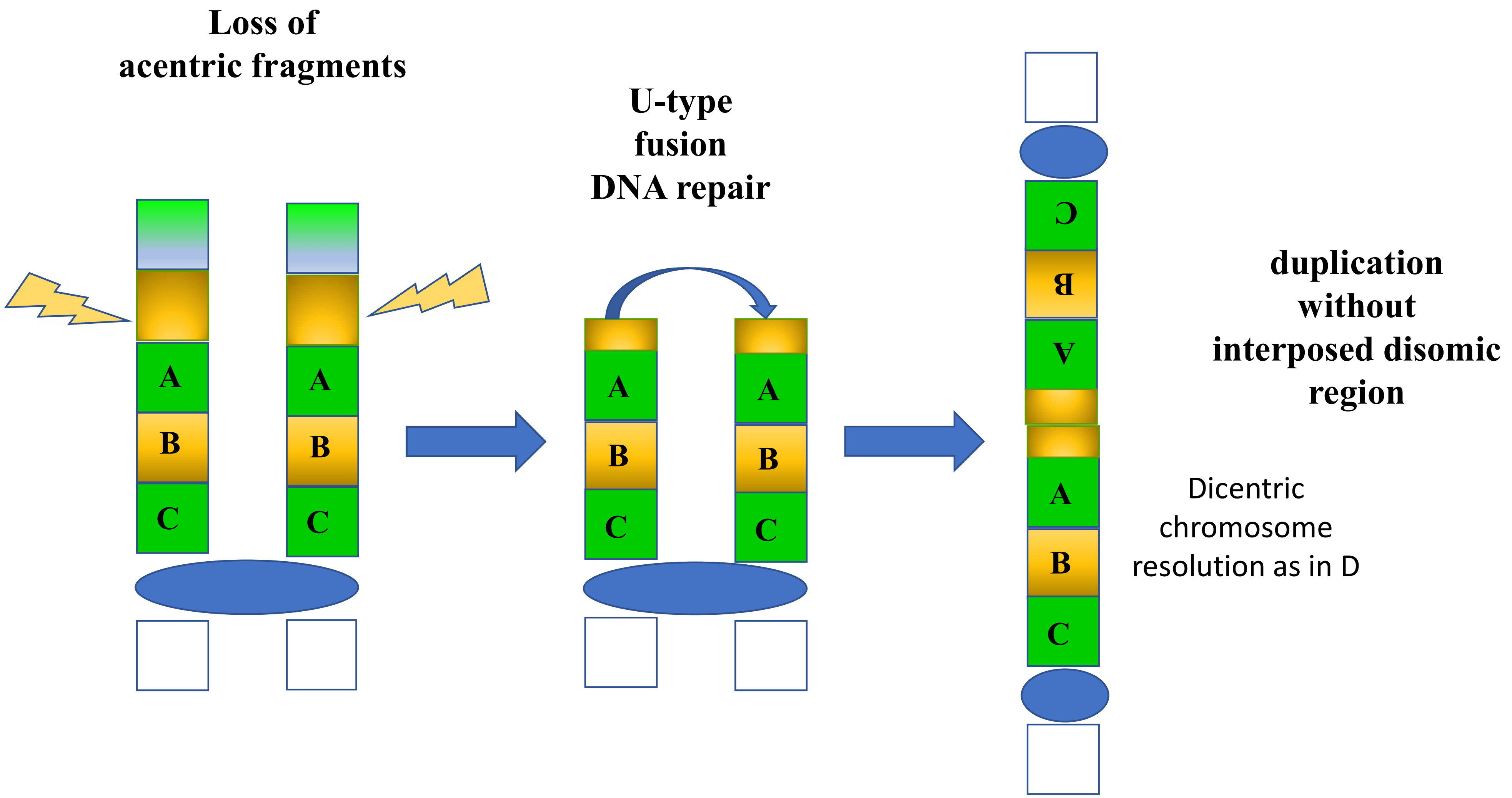
Loss of
acentric fragments
A
B
C
A
B
C
U-type
fusion
DNA repair
A
B
C
A
B
C
duplication
without
interposed disomic
region
Dicentric
chromosome
resolution as in D

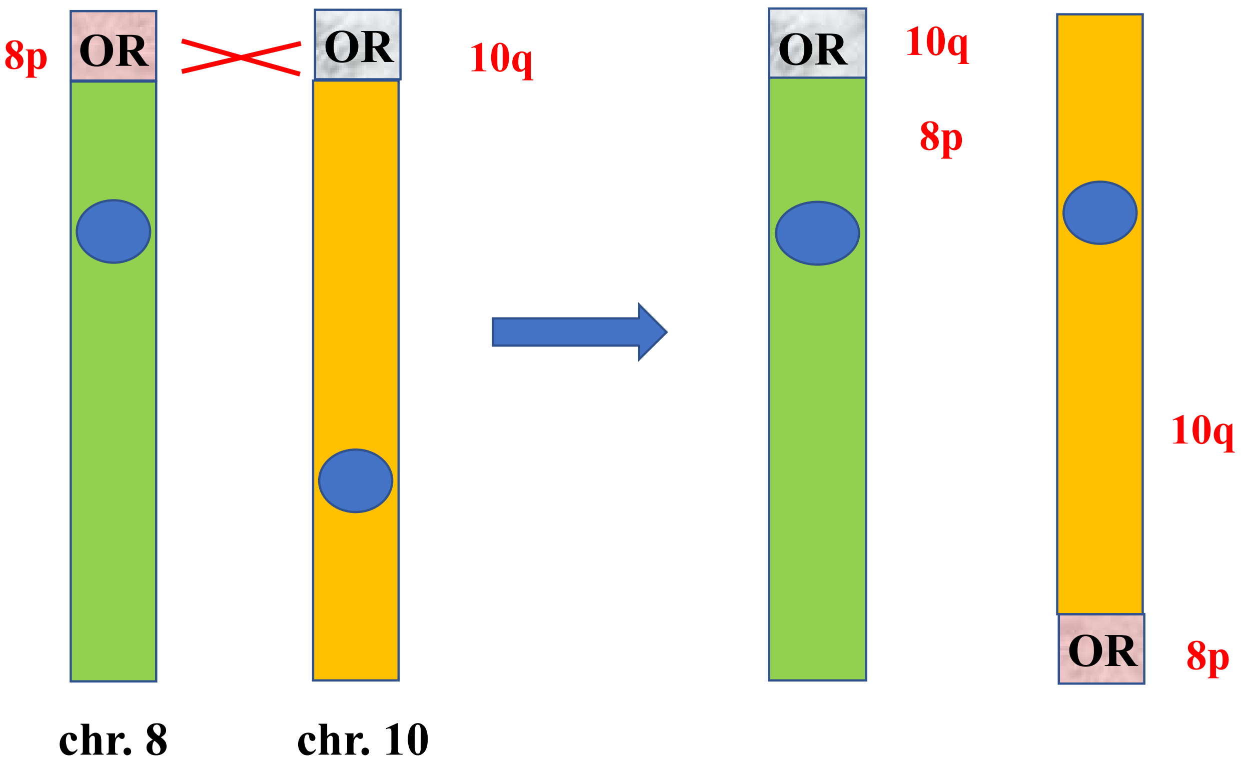

maternal reciprocal translocation

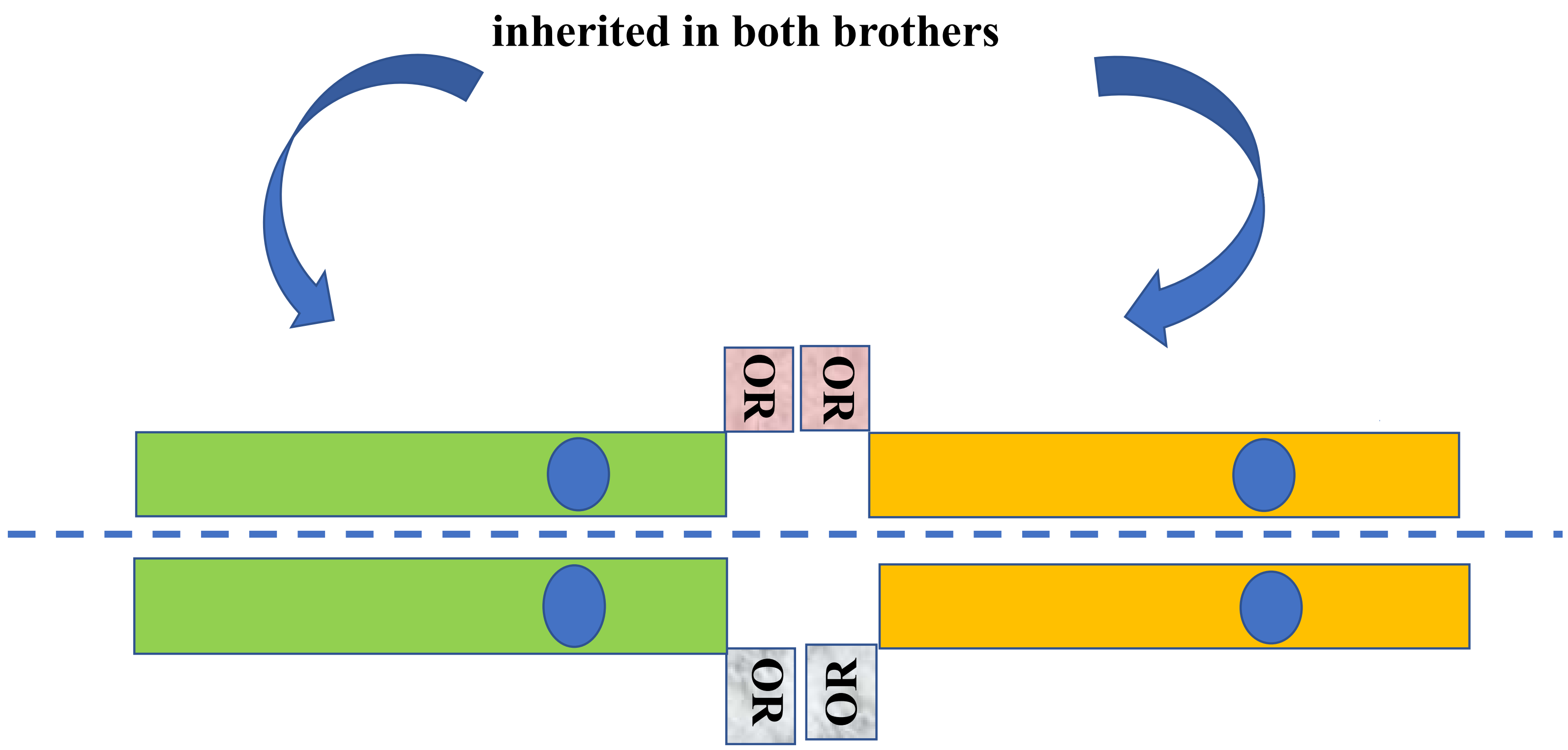

meiotic pairing and segregation
adjacent 1
